# Microarray-Based Sketches of the HERV Transcriptome Landscape

**DOI:** 10.1371/journal.pone.0040194

**Published:** 2012-06-28

**Authors:** Philippe Pérot, Nathalie Mugnier, Cécile Montgiraud, Juliette Gimenez, Magali Jaillard, Bertrand Bonnaud, François Mallet

**Affiliations:** 1 Joint Unit Hospices Civils de Lyon, bioMérieux, Cancer Biomarkers Research Group, Centre Hospitalier Lyon Sud, Lyon, France; 2 BioMérieux, Data and Knowledge Laboratory, Marcy l’Etoile, France; University of Poitiers, France

## Abstract

Human endogenous retroviruses (HERVs) are spread throughout the genome and their long terminal repeats (LTRs) constitute a wide collection of putative regulatory sequences. Phylogenetic similarities and the profusion of integration sites, two inherent characteristics of transposable elements, make it difficult to study individual locus expression in a large-scale approach, and historically apart from some placental and testis-regulated elements, it was generally accepted that HERVs are silent due to epigenetic control. Herein, we have introduced a generic method aiming to optimally characterize individual loci associated with 25-mer probes by minimizing cross-hybridization risks. We therefore set up a microarray dedicated to a collection of 5,573 HERVs that can reasonably be assigned to a unique genomic position. We obtained a first view of the HERV transcriptome by using a composite panel of 40 normal and 39 tumor samples. The experiment showed that almost one third of the HERV repertoire is indeed transcribed. The HERV transcriptome follows tropism rules, is sensitive to the state of differentiation and, unexpectedly, seems not to correlate with the age of the HERV families. The probeset definition within the U3 and U5 regions was used to assign a function to some LTRs (i.e. promoter or polyA) and revealed that (i) autonomous active LTRs are broadly subjected to operational determinism (ii) the cellular gene density is substantially higher in the surrounding environment of active LTRs compared to silent LTRs and (iii) the configuration of neighboring cellular genes differs between active and silent LTRs, showing an approximately 8 kb zone upstream of promoter LTRs characterized by a drastic reduction in sense cellular genes. These gathered observations are discussed in terms of virus/host adaptive strategies, and together with the methods and tools developed for this purpose, this work paves the way for further HERV transcriptome projects.

## Introduction

The concept of endogenous retroviruses (ERV) dates back to the 1970’s and particle-budding observations in the years that followed have gradually provided evidence that mammal genomes serve as reservoirs for retroviral elements [1], [2], [3]. Later, the sequencing of distinct species unveiled the contribution of the ERV subset within transposable elements (TE), and highlighted in particular a similar proportion of retrovirus-like sequences in human and mouse genomes (8–10%) [4], [5], [6], [7]. The endogenous retrovirus pool is thought to originate from ancestral and independent infections within the germ line [8], [9], before complex re-infection, retro-transposition, propagation and error-prone steps occurred during evolution. In humans, the definition of at least 31 HERV families is commonly accepted in reference to putative ancestors [10]. As a result, each family contains tens to thousands of distinct loci scattered throughout the human genome.

To date, all the HERV elements that have been characterized are defective for viral replication. Nevertheless, the discovery that some HERV proteins may contribute to biological events has quickly generated interest in open reading frame (ORF) sequences. The Syncytin-1 and Syncytin-2 envelope glycoproteins are encoded by full-length HERV sequences belonging to the HERV-W and HERV-FRD families, respectively and, through cell differentiation mechanisms, these proteins are presumably essential for human placentation (reviewed in [11]). Syncytin-1 is also associated with epithelial cancers [12], [13] and was recently detected in the peripheral blood of leukemia and lymphoma patients [14]. Among the HERV-K HML-2 family, full-length proviruses can encode either Rec or Np9 proteins, which are known to interact with cellular partners and ultimately may affect cancer signaling pathways [15], [16], [17], [18]. Although HERVs match the self-antigen concept, the immune response directed against HERV-K HML-2 Env and Gag proteins is remarkably detectable in the blood of patients with seminoma up to six months before diagnosis [19], [20], [21], [15] and thus may form a basis for molecular tools for early germ cell tumor detection.

However, the role of HERV in human biology should not only be reduced to ORF and putative coding genes (in reference to oncoviruses) since TE also contribute to genome plasticity. Duplication of Alu sequences, recombination, and transduction of LINE elements may have led to multigenic families, gene duplication and exon shuffling [22], [23], [24]. In particular, the long terminal repeat (LTR) sequences of HERV elements may be the source of inter-element recombination phenomena resulting in chimerical proviruses, tandem structures and solitary LTRs [25], [26], [27]. Current estimates indicate that the human genome harbors around 200,000 HERVs (excluding MaLR), mainly composed of sequences resembling LTRs [5], [28], [29]. Taking into account that LTRs exert natural transcription functions within a retrovirus, it is likely that some have now retained the potential to act as regulatory elements [30], [31].

In this context, many studies have established a role for LTRs as a promoter [32], [33], [34], [35], [36], [37], [38], bidirectional promoter [39], [40], enhancer [41], [42], polyadenylation signal [43] and antisense transcript negative modulators [44] of cellular genes in different biological contexts (for a full review see [45]). On the basis of serendipitously and case-by-case identifications, knowledge of functional interactions between HERV elements and cellular environment has gradually grown and is increasingly based on systematic approaches. As different works now estimate that more than 50% of human genes use alternative promoters [46], [47], the importance of accurately identifying distinct HERV elements in transcriptome-wide studies, documenting their expression in a variety of biological contexts and finally assessing the question of their regulation in connection with their genomic environment is a strong argument for the need for a HERV transcriptome project [48].

Over the last 10 years, most of the attempts for HERV expression measurement used RT-PCR techniques either to focus on a specific locus [49], [50], [51], [52], [53] or to evaluate general trends within HERV families or genera [54], [55], [56], [57]. Yet the inherent limitations in the development of reliable PCR systems to discriminate individual HERV elements in a holistic approach require fairly laborious work [58], [48]. On the other hand, methods based on expressed sequence tags (ESTs) provided a more comprehensive view of the HERV transcriptome but generally ran into trouble for identifying the unique genomic source of expression [59], [60].

We previously developed an early high-density microarray generation dedicated to the HERV transcriptome, given promising results in terms of tropism and individual locus identification notwithstanding high risks of cross-reactions [61]. Following this attempt, in this work, we introduced a new methodology suitable for repeated element probe design aiming to minimize cross-reactions. At the same time, we expanded the content of the chip to 6 HERV families: HERV-W, HERV-H, HERV-E 4.1, HERV-FRD, HERV-K HML-2 and HERV-K HML-5, providing the user with a collection of 2,690 distinct proviruses (complete or partial) and 2,883 distinct solo LTRs ready for expression monitoring. Additionally, independent probesets within U3 and U5 regions made it possible to assign a function (i.e. promoter or polyA) to 1,513 LTRs. We used this next generation microarray to gain insights into the HERV transcriptome using a composite panel of 40 normal and 39 tumor RNA samples. We found that HERV expression patterns are highly dependent on tissue type and differentiation state and accordingly we established a list of potential HERV biomarkers. We also identified 326 and 209 LTRs with putative promoter and polyA activity, respectively, and highlighted extensive operational determinism for active LTRs. We finally emphasized the trend for promoter LTRs to be associated with an upstream 8 kb zone characterized by a poor sense cellular gene density, compared to silent and polyA LTRs. Taken together, these data allowed us to discuss the adaptive relationship between viruses and host and to prepare a first draft of the HERV transcriptome that could help renew the role of the HERV repertoire in the context of what was improperly named ‘junk’ DNA.

## Results

### Detection of the HERV Transcriptome

We constructed a database grouping 10,035 distinct HERV elements that belong to 6 HERV families (Table 1*a*), and we used it as an input collection for the design of a new and suitable HERV-dedicated microarray, called HERV-V2. For this purpose, we developed a scoring function which assesses the ability of a 25-mer probe/target pair to hybridize in Affymetrix-based technology format. This function, referred to as EDA+, allowed us to exclude candidate probes that did not meet specificity criteria. The resulting HERV-V2 chip can discriminate 5,573 distinct HERV elements (23,583 probesets) that can reasonably be assigned to a unique genomic position, including functional U3/U5/gag/pol/env parts, either for provirus structures or solo LTR elements (Table 1*b*).

**Table 1:** Detection of the HERV transcriptome.

Repertoire	Elements^e^	HERV-W	HERV-H	HERV-E 4.1	HERV-FRD	HERV-KHML-2	HERV-K HML-5	Total
**Genome** a	**solo LTRs**	**464**	**1079**	**158**	**1259**	**1000**	**87**	**4047**
	**complete or partial** **proviruses**	**823**	**1492**	**455**	**349**	**2685**	**184**	**5988**
	5′ LTRs^d^	128	1036	41	36	52	22	1315
	3′ LTRs^d^	219	1062	39	45	2482	22	3869
	gag^d^	199	1093	246	88	117	126	1869
	ppol^d^	234	0	0	96	0	0	330
	pol^d^	0	1315	330	75	155	147	2022
	env^d^	240	1173	67	154	2548	97	4279
**Chip** b	**solo LTRs**	**432**	**553**	**120**	**1189**	**512**	**77**	**2883**
	**complete or partial** **proviruses**	**304**	**1354**	**427**	**218**	**215**	**172**	**2690**
	5′ LTRs^d^	120	444	29	33	29	18	673
	3′ LTRs^d^	171	485	29	43	85	19	832
	gag^d^	162	787	228	80	85	125	1467
	ppol^d^	222	0	0	0	0	0	222
	pol^d^	0	1154	307	35	93	135	1724
	env^d^	205	513	63	127	66	97	1071
**Transcriptome** c	**solo LTRs**	**100**	**209**	**30**	**251**	**199**	**19**	**808**
	**complete or partial** **proviruses**	**101**	**587**	**91**	**39**	**75**	**17**	**910**
	5′ LTRs^d^	26	154	10	8	10	4	212
	3′ LTRs^d^	43	182	10	11	35	7	288
	gag^d^	12	202	51	4	15	4	288
	ppol^d^	8	0	0	0	0	0	8
	pol^d^	0	170	28	1	9	2	210
	env^d^	49	71	5	16	12	2	155

a Number of distinct genomic HERV loci included in HERV database HERV-gDB3. The database contains 6 HERV families with unequal input. The search for distinct elements belonging to each family is performed by systematic BLAST genome coverage, allowing a maximum 20% divergence with prototype elements.

b Number of distinct genomic HERV loci present in the chip. Each element of the database is processed through home-made EDA+ algorithm to find probes that match optimal hybridization criteria. The candidate probes are then checked against the entire human genome (NCBI 36/hg18) using the KASH algorithm to control their cross-hybridizing ability and non-specific sequences are removed. Probes are ultimately assembled into probesets to discriminate individual genomic HERV sequences. Differences between database and chip mark the success in designing HERV-specific probes and probesets. For clarity, the probeset content is not detailed.

c HERV transcriptome results: number of active elements in all tissues tested. After the experiments were normalized using the COMBAT method and an arbitrary positive threshold was applied (value = 100), elements that are active in at least one tissue are enumerated.

d Subsets of complete or partial proviruses.

e One element can be composed of several probesets.

As an initial view of the HERV transcriptome, we performed a study based on a diversified panel composed of both normal and tumor tissues, including testis, colon, ovary, prostate, breast, uterus, lung and placenta samples. Noteworthy, all samples except placenta are matched normal/tumor tissues obtained from the same individual. The set of data revealed transcriptional activity for 1,718 distinct HERV elements (Table 1*c*), which is about one third of the HERV-V2 chip contents and may suggest a similar proportion of active elements among the human genome. We then sought (i) to determine whether HERV expression varies depending on the tissue and thus follows tropism rules or not, (ii) to find out the extent to which HERV elements are sensitive to the state of differentiation and may serve biomarker research, (iii) to gain insight into transcriptional mechanisms in the light of genomic environment and (iv) to reinforce the comprehensive role of the HERV repertoire in our biology.

### Characterization of the HERV Transcriptome

#### Tropism of Active HERVs

**Figure 1:**
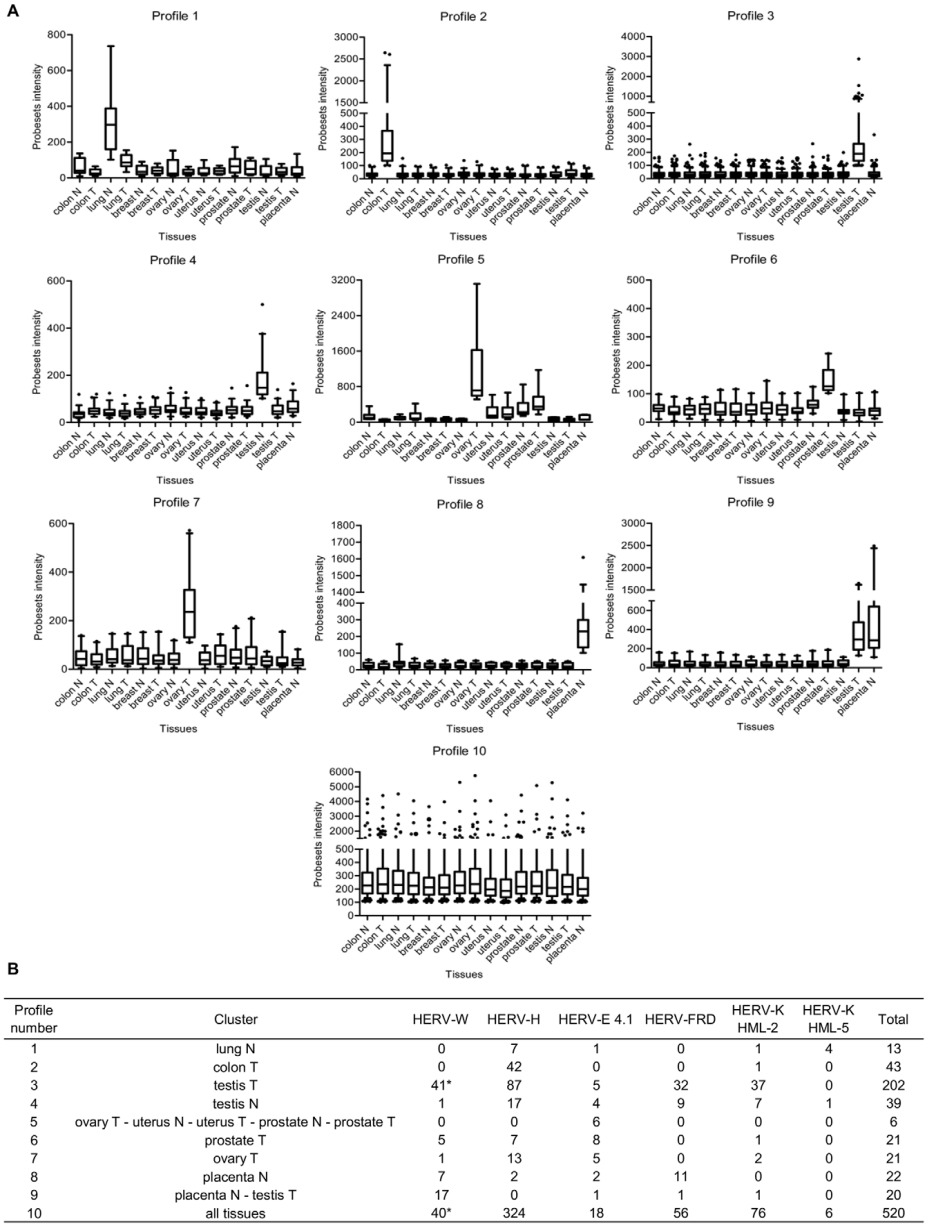
Tropism of active HERVs. (**A**) Active probesets ‘cluster’ into 10 expression profiles. The final number of profiles is estimated after iterative corrections combining Euclidean partitioning algorithm and fine manual adjustment steps. Box plots indicate the distribution and the median of probeset intensity, whiskers are 5–95 percentiles, dots show outliers. The order of profiles is not important. (**B**) Profile description. Each profile refers to a specific cluster of tissues, and involves a number of probesets detailed by families. By definition, a probeset is classified in a unique profile, except for the asterisk (*) where a single probeset is willingly shared by both profiles 3 and 10.

To determine whether the nature of a tissue affects HERV expression, we classified active probesets according to their expression pattern. Although a large proportion shows either no expression or weak unclassifiable signals (data not shown), 10 expression profiles were obtained from partitioning clustering (Figure 1A). The final sizes and the resolving power vary from one profile to another in accordance with data structure. Among the 10 profiles, 2 main types should be distinguished, whether the profile involves only one tissue, or more than one. In profiles 1, 2, 3, 4, 6, 7 and 8, the probesets have a single-tissue expression and consequently can be considered to be tissue-state sensitive. On the other hand, profiles 5, 9 and 10 are dedicated to active probesets expressed in more than one tissue (even being expressed in all tissues such as in profile 10), and thus must reflect a more complex tropism. A detailed list of all HERV loci composing the groups of expression, including genomic coordinates, is provided in Table S3.

In an attempt to unveil a particular behavior in such expression patterns, the number of probesets is summarized taking into account the 6 HERV families (Figure 1B). Interestingly, some profiles coincide with a predominant family representation. This is the case among the colon tumor group (profile 2) where the HERV-H family is almost exclusively present, as well as for profile 5 that is entirely described by the HERV-E 4.1 family, and for profile 9 which mostly involves HERV-W probesets.

#### Differential Expression Associated with Tissue State Changes

**Figure 2:**
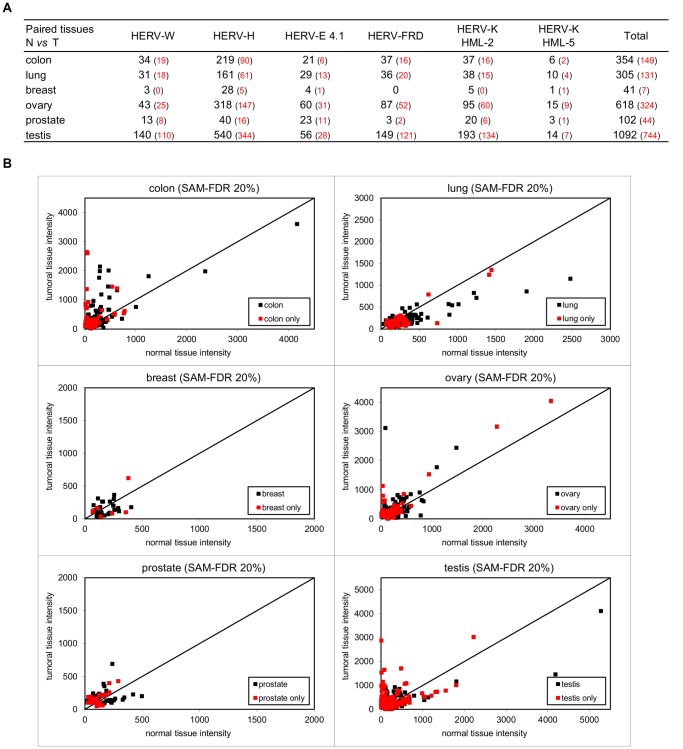
Differential expression induced by tissue state changes. (**A**) Pairwise analysis. For each paired tissue, the SAM-FDR method is applied and leads to the identification of a number of probesets that show significant differential expression (FDR  = 20%). The red number in brackets indicates how many differential expressed probesets are specific to the tissue. Uterus normal versus tumor comparison gives no result and consequently does not appear in the table. (**B**) Scatter plots of expression values. Normal versus tumor normalized expression values of differential expressed probesets are draw for each tissue pair. The statistically significant absence of differential expression is represented by the diagonal line (y = x). Red plots refer to tissue-specific probesets (previously mentioned using red numbers in bracket in Figure 2A).

To gain insight into the variation of expression associated with tissue state, we performed supervised statistical analysis in pairwise tissues using the SAM method with FDR correction. In order to easily compare the results from the different tissues, we used a high and constant false discovery rate (FDR = 20%). Each paired tissues (i.e. normal tissue versus adjacent tumor tissue) gives an independent number of differentially expressed probesets, ranging from zero (in uterus, data not shown), to 1,092 in testis (Figure 2A). Additionally, the lists are compared to highlight tissue-specific probesets.

To get a better view of the relevance of the results obtained with the SAM-FDR procedure, we drew scatter plots of normal versus tumor expression values for each tissue pair (Figure 2B). The density of plots, the relative amount of tissue-specific probesets (in red) as well as the deviation from the reference straight line together serve to distinguish valuable probesets from non-relevant results. Thereby colon, testis and in a lesser extent ovary and lung involve numerous probesets showing both significant variation of expression and tissue specificity. A list of all the HERV loci that show differential expression together with their associated genomic coordinates is provided in Table S3.

#### LTR Functions

**Figure 3:**
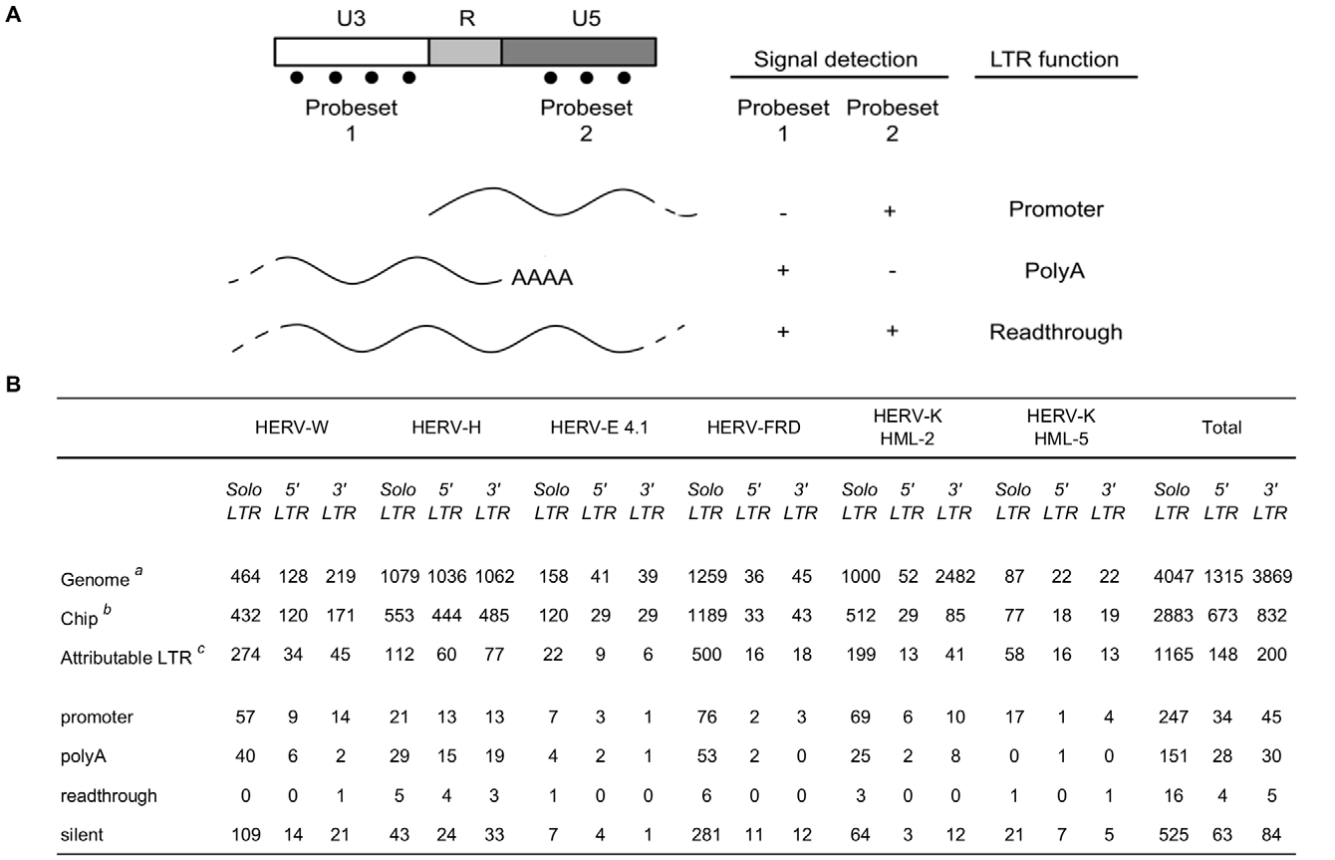
LTR functions. (**A**) Schematic view of LTR structure and associated theoretical transcription events. Top to bottom: the LTR is a natural or alternative promoter when the transcription starts between U3 and R/U5; the LTR ends an upstream transcription event by the addition of polyA tail at the end of the R region; the transcription passes through the LTR with no incidence in the progression of the polymerase, which results in the detection of U3, R and U5 transcripts. Rules for function assignment are *promoter*: U3−/U5+; *polyA*: U3+/U5-; *readthrough*: U3+/U5+ and *silent*: U3−/U5-; with expression levels: + >100; - <50. Expression levels between 50 and 100 delineate an indeterminate grey area where a function is assigned if the ratio between U3 and U5 is greater than 4 (for instance U3 = 80 and U5 = 321 is counted as promoter). Otherwise, the LTR function is declared to be unknown. (**B**) Assignment of functions. *^a,b,c^* Loss of information from HERV database to understandable functions. *^a^* Summary of Table 1*a* *^b^* Summary of Table 1*b* *^c^* Enumeration of LTRs whose function is attributable, i.e. defined as LTR combining both complete structure on the genome and existing probesets on the chip, that can ultimately allow a discrimination between U3 and U5 expression signals.

To approach the question of HERV transcription mechanisms, we focused on LTR signals. In the context of the distribution of a substantial number of retroviral sequences throughout the human genome, we assessed the question of LTR functions regardless of the original provirus structure. Based on the fact that one LTR can theoretically assume different functions depending to its environment, we systematically tested whether the transcription initiates, ends within the LTR, or passes through the element with no incidence on the transcription process (Figure 3A).

We used the dichotomy of signals acquired from probesets distributed along U3 and U5 regions to assign the functions: U3-associated negative signals and U5-associated positive signals for promoter, U3 positive signals and U5 negative signals for polyA, U3 and U5 double positive signals for readthrough. Loci which exhibited double negative U3 and U5 signals were classified as silent (expression level cutoff = 50 for negative signal). Expression levels between 50 and 100 delineate an indeterminate grey area where a function is assigned only if the ratio between U3 and U5 signals is greater than 4 (see legend of Figure 3A for details). Due to the general LTR sequence homology and the large share of partial and complex structures, only a small fraction of LTRs meet the requirement to infer a function. These LTRs are referred to as ‘attributable LTR’ - aLTR in the text - in Figure 3B and represent one third of the chip LTR content.

Of all the tissue samples tested, we finally identified a total of 326 distinct autonomous ‘promoter’ LTRs (21% of aLTR) and 209 distinct ‘polyA’ LTRs (13% of aLTR). Very surprisingly, there is no overlap between these two LTR lists except one which belongs to the grey area. This highlights that active LTRs cannot switch from promoter to polyA function even if the tissue changes, which we will refer to as operational determinism. To enhance this opinion we repeated LTR function analysis using a completely different set of data coming from cell lines that were subject to chemical and oncogenic transformations (data not shown). Using 6 different cell lines, we found a parallel list of 67 promoter and 46 polyA LTRs and still no overlap between promoter and polyA LTRs exists. Moreover, the cell culture-derived list closely matches the results from tissues: among the 113 (67 promoters +46 polyA) active LTRs unambiguously characterized from the cell culture, only 7 LTRs did not intersect with the 535 LTR list (326 promoters +209 polyA) characterized from tissues. This means that less than 2% of new characterizations have been gained by diversifying biological records. Consequently, this result prompts us to conclude that we have delineated a stable pool of active and functional LTRs.

Most of the function characterizations concern solo LTRs with 247 promoter solo LTRs and 151 polyA solo LTRs (26% of aLTR), but the function distribution seems to be unbalanced between families: although there is generally a low number of output cases, we observed for instance that the HERV-K HML-5 family has no occurrence of polyA solo LTRs, and we noted that the HERV-K HML-2, HERV-W, HERV-E 4.1 and HERV-FRD families have more promoter solo LTRs cases than polyA solo LTRs cases, whereas the HERV-H family, on the contrary, seem to involve a greater amount of polyA solo LTRs compared to promoter solo LTRs. Focusing on provirus structures, we identified 34 promoter 5′LTRs and 30 polyA 3′LTRs. In some cases, we associated both 5′ promoter and 3′ polyA activities within a given provirus. We also discovered 45 promoter 3′LTRs and 28 polyA 5′LTRs.

Besides these 34% comprehensive active aLTR, we also showed that a high proportion of the LTR population always remains silent (672 cases; 44% of aLTR). In addition to that, we identified a smaller number of readthrough LTRs (25 cases; <2% of aLTR).

#### Validation Analyzes

We first compared our results with previously published data focusing on the HML-2 family as this family has been widely studied using various methodologies. We included data derived from EST study [59], genomic repeat expression monitoring (GREM) for experimental genome-wide identification of promoter-active repetitive elements [62], PCR-sequencing [48] and array-based approaches [61]. Of the 327 HML-2 elements we analyzed, 25 elements were shared by at least one of the previous studies (Table S4). On this subset, Affymetrix-based format analysis gave 64% and 63% correlation with the EST approach and the PCR-sequencing-based study, respectively. A poor correlation of 19% was observed with GREM.

To confirm the tropism of active HERV, we then tested whether the elements we classified within expression groups using HERV-V2 correlate with tissue-related EST libraries. A fairly clear enrichment of the expected EST population was observed in the case of colon, ovary and placenta and can also reasonably be claimed in the case of testis taking into account that testis-associated HERV sequences were initially distributed into 3 expression groups (Table S5). In contrast, results depicted for lung and prostate were not supported by ESTs, probably due to an overall less-pronounced expression level of related HERV elements. We then picked 33 candidate loci and designed PCR primer pairs which were evaluated for sequence specificity using high resolution melting and sequencing (see Materials and Methods, RT-PCR). Eighteen highly specific primer pairs corresponding to 8 loci were eventually selected and tested on samples (Table S6). An overall good correlation of 0.926 (min 0.606; max 0.998) between arrays and RT-PCR was observed, essentially confirming the attributed tropism (Figure S2). Nevertheless, unexpected expression was found twice for two HERV-H proviruses, in cancerous colon in addition to the expected expression in tumor testis, and in cancerous ovary in addition to the expected expression in tumor colon.

The LTR functions were assessed using U3 versus U5 RT-PCR assays. Using this strategy, we previously validated the promoter function of 6 loci expressed in testicular cancer identified by the first version of the HERV microarray [61], which was confirmed in this study (data not shown). Such a strategy was used again and confirmed two new tropism-related promoters (200261_w and 1100414_2) as well as one ubiquitous promoter (2000062_2) as presented in Figure S3. Then, to broaden the scope of such analysis, we sought to confirm LTR functions by analyzing the U3 versus U5 distributions of LTR-associated ESTs for a subset of the HERV-W family consisting of 21 proviruses and 110 solo LTRs or LTRs associated with truncated proviruses. We focused on the HERV-W family because it contains the ERVWE1 domesticated locus in which the 5′LTR promoter and the 3′LTR polyA functions have been exhaustively demonstrated [63], [64], [65], [66], [61]. Results are depicted in Table S7 and alignments are provided in Figure S4 **.** In brief, only 17 loci among the 131 loci analyzed exhibited significant LTR-associated ESTs and only 16 loci are ultimately interpretable. 8 EST-deduced functions (7 promoters, 1 polyA) were consistent with those we identified following microarray results. Two other promoter functions were compatible with an upstream alternative transcription initiation site (see locus 1200505_w and locus 600462_w in Table S7). One additional promoter function was plausible (locus 400207_w) although an alternative splicing event excluding U3 could be involved. Finally, one readthrough identified using microarray (locus 700126_w) could be classified either as polyA or readthrough with regard to EST data. Four comparisons were discrepant, opposing readthrough to promoter function, and putatively identifying a removal of the U3 region in mRNAs due to a splice occurrence. Altogether, the overall correlation between array and EST-deducible functions ranged between 50% and 75%.

#### Influence of the Genomic Environment

**Figure 4:**
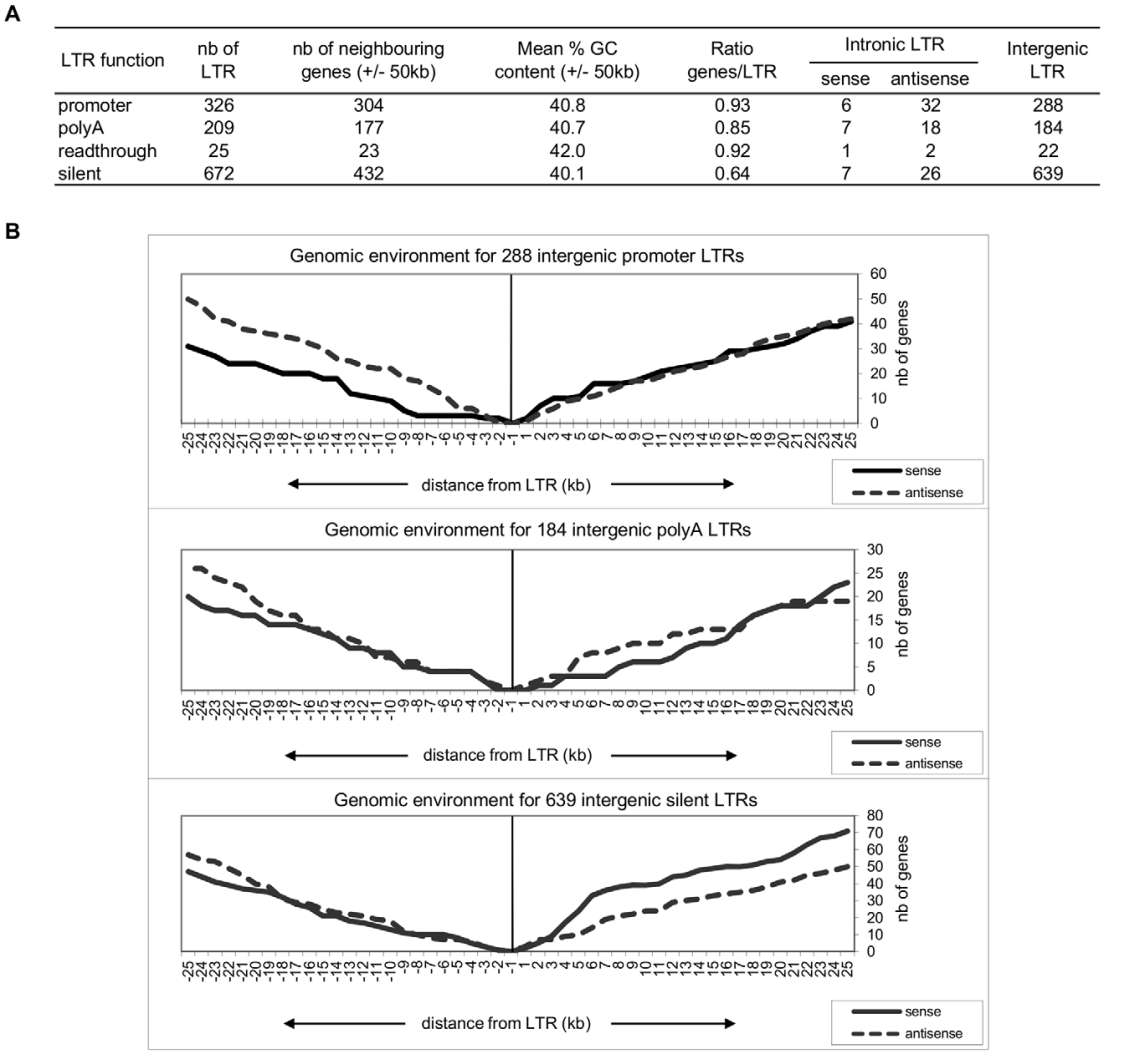
Genomic environment of functional and silent LTRs. (**A**) Overview of genomic and chromatin composition (%GC) of functional LTR neighborhood. For all promoter, polyA, readthrough and silent LTRs, the number of neighboring genes in the surrounding +/−50 kb is obtained from NCBI 36/hg18 using annotations from the RefGene table (UCSC), then the DNA sequences are extracted *in silico* for %CG content calculation. The table includes the number of intronic functional LTRs, defined as LTRs that overlap gene limits (NCBI 36/hg18 RefGene table), and ends with the number of intergenic LTRs. *Sense*: LTR and gene are in the same orientation; *antisense*: LTR and gene are in opposition. (**B**) Genomic environment for intergenic functional LTRs. Genes in the same orientation (*sense*) or in opposition (*antisense*) with the LTRs are counted in the case of promoter, polyA and silent intergenic LTRs. Read-through LTRs are not included as their number, which is too low, does not fit with the representation. Vertical bar centered on zero should be interpreted as an ellipse of the LTR sequence. Away from the bar, the cumulative gene occurrence is shown up to +/−25 kb starting from the LTR limits. Curve tendencies beyond 25 kb do not change significantly and are not represented.

We extended our investigation to the genomic environment encompassing the newly-identified functional and silent LTRs. For each LTR, we performed a search for gene presence and %GC content in the surrounding 50 kb, starting from the limits of the LTR. When the position of the LTR overlaps with the position of the gene, the LTR is counted as intronic. The total number of neighboring genes normalized by the initial number of LTRs gives a gene density ratio detailed for each category (Figure 4A). The gene density ratio is almost 1.5 times higher for active LTRs than for silent LTRs although the %GC barely varies. Meanwhile, the proportion of intronic LTRs is largely in favor of antisense representation for all categories of LTRs.

The case of intergenic LTRs is subject to a more detailed description in Figure 4B. For promoter, polyA and silent LTR groups, a cumulative gene distribution function is drawn upstream (5′) and downstream (3′) of the LTR limits (vertical bar) emphasizing whether the genes found away from the LTR have the same orientation as the LTR (sense) or not (antisense). This revealed a strikingly low occurrence of genes in sense orientation up to 8 kb upstream of promoter LTRs while the upstream 8 kb for silent and polyA LTRs shows no difference regarding the gene orientation. Besides, the downstream environment also appears to be linked to the LTR function but in a kind of mirror situation in which the sense genes occurrence apparently rises faster than for antisense genes in the downstream 8 kb zone of silent LTRs compared to promoter and polyA LTRs.

#### Lessons Learned from the HERV Transcriptome

**Figure 5:**
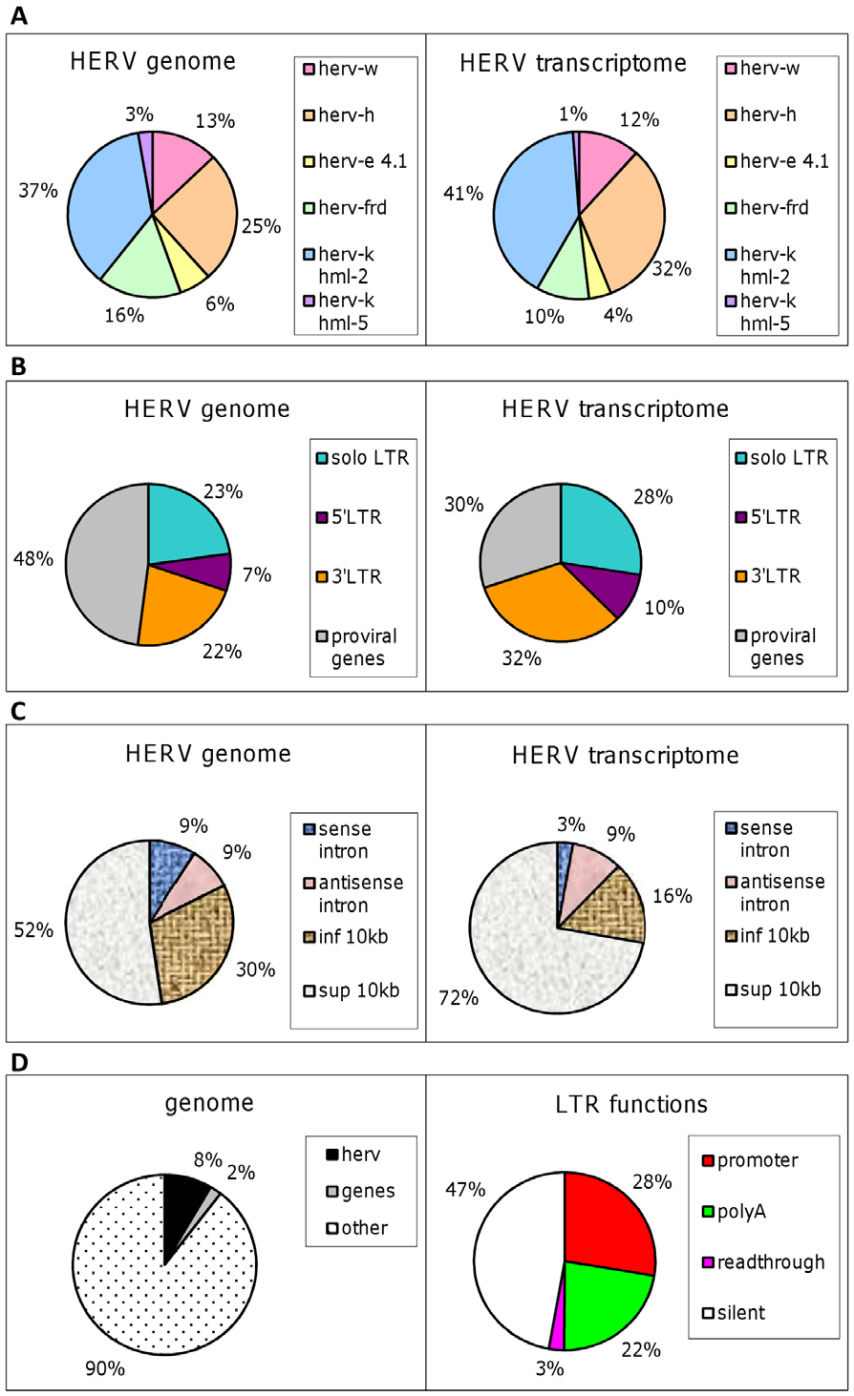
Genomic and transcriptomic projections of the HERV repertoire. (**A**) HERV families. The 6 HERV families studied in this work are voluntarily depicted as 100% of HERV human genome, in the proportions described in Table 1*a*. The transcriptome picture is obtained from results detailed in Table 1*c* after applying a correction factor that takes into consideration chip content in Table 1*b*. (**B**) HERV structures. Solo LTR and proviruses account for 100% of the HERV genome in the proportion described in Table 1*a* *.* The transcriptome part is based on Table 1*c* after correction taking into consideration the chip content presented in Table 1*b* *.* (**C**) HERV environment. A systematic search for genomic environment is performed for elements present in the HERV database (genome) and the active elements described in Table 1*c* (transcriptome). The proportion of the 3 types of LTR is based on Table 1*a* and the transcriptome from Table 1*c* after correction using Table 1*b* *.* (**D**) The role of junk DNA. HERV sequences represent approximately 8% of the human genome. The graph of LTR functions is based on Figure 3B after a correction based on the number of attributable functions and chip content.

The different results were finally used to construct a comparative view of HERV genome and transcriptome. To achieve this goal, we used the term ‘HERV genome’ to refer to the entirety of our HERV genome database content (i.e. 6 HERV families), and we opposed the HERV transcriptome resulting from our experiments (Figure 5). Since the HERV-V2 content reflects the success in designing specific probes and probesets, which varies from one family and one element to another, we had to apply correction factors to raw transcriptome results. Accordingly, the following outcome must be regarded as an extrapolation.

The first observation tends to show there is no difference between the contribution of each family to genome and transcriptome sharing (Figure 5A). However, the transcription seems to be impacted by the structure of HERV elements in a trend that aims to reduce proviral gene expression (30% to 16%) (Figure 5B). More flagrantly, the genomic environment appears to exert a major influence as the expression of HERV elements that map close to human genes (<10 kb) is twice constricted and, at the same time, the expression of intronic HERV sequences in sense orientation reduces dramatically (9% to 3%) (Figure 5C). Focusing on LTRs and regardless of the tissue tested, almost 50% of the LTR elements remain silent, while active LTRs are roughly equally divided between promoter and polyA functions (Figure 5D). We also identified very few cases of readthrough LTRs (3%).

## Discussion

### HERV Transcriptome Views

We provided a microarray-based description of the HERV transcriptome based on the analysis of a set of cancerous and non-cancerous tissues that reflect a range of diversity. Different works have been conducted to discover the contribution of HERV to the human transcriptome [38], [60], [59], [62], [48], [61]. In this study we identified 1,718 active HERV elements suggesting that about 30% of the retroviral sequences spread across the genome are transcribed. Despite the fact that it is usually thought that HERVs colonize the genome and consequently are tightly controlled to avoid gene disruption [67], our observation of a substantial basal HERV transcriptional activity is partly supported by others. In 2008 Conley *et al*. analyzed high-throughput expression data to claim that transcribed HERV sequences correspond to 1.16% of the human genome sequence [38], which would mean that approximately 15% of HERV sequences are active. Previous analyses of HERV activity based on ESTs led Oja and colleagues to estimate that 7% of the HERV sequences are transcribed [68]. More generally, the fact that the human genome might be more or less pervasively transcribed, including sequences previously thought to be silent, was a key outcome of the ENCODE pilot project and led to the proposal of the ‘warehouse’ concept for natural selection [69]. This suggests how HERV may regulate human transcription on a large scale.

EST data appear to be insufficient to describe the transcriptional activity of HERVs and therefore to unambiguously characterize promoter functions, as previously discussed [60], [48]. Moreover, for the most active HERV elements, Oja reported hundred to thousand-fold over-representation of *pol* and *env* regions (as opposed to LTRs). Such poor EST detection in either 5′ or 3′ LTRs could be due to the nature of the EST methodology which may be sensitive to low level of expression or end-location of secondary structured LTRs on mRNA or even the occurrence of polypurine tracks within retrovirus genes [70]. Notably, EST strategy failed to identify ERVWE1/Syncytin-1 3′LTR as a polyA signal as discerned using HERV-V2, although the full-length Syncytin-1-containing polyadenylated cDNA has been isolated [63]. Nevertheless, focusing on the 22 well-described HML-2 elements we shared with the EST study conducted by Stauffer, we obtained a correlation of 64%. Similarly, the circumscribed EST analysis conducted on HERV-W elements confirmed up to 75% of our promoter elements.

### HERV Tropism and Implication in the Biomarkers Field

The HERV transcriptome presented herein was generated using a set of tissues selected in order to support the hypothesis that individual HERV can serve the biomarker field. Among cancerous and non-cancerous tissues, we characterized expression patterns supporting that testis and placenta are privileged places of HERV expression. Syncytin-1, a functional envelope glycoprotein belonging to the HERV-W family, is expressed in the placenta and in the testis [63], [71], [49], [61]. Syncytin-2, a member of the HERV-FRD family, takes part in the placenta expression cluster [72], [49], [73] and numerous envelope and capsid elements related to the HERV-K HML-2 family formed the testicular tumor group as described previously [2], [74], [75], [19], [76]. In a recent work, we reported the expression of 6 HERV-W elements in testicular tumor using an early version of HERV chip [61]. This second generation of the HERV chip allowed to confirm the overexpression of 5 out of 6 elements (the 6^th^ locus belongs to the grey area as defined above) and, at the same time, we identified numerous new HERV-W elements specific to the testicular cancer sample with high expression levels. The association of HERV-H elements with colon cancer [59], [77], [78], [79] and the finding of HERV-E 4.1 sequences in a group composed of prostate, uterus and ovary samples has also been reported [61] and is confirmed here. Taken together, these findings argue in favor of non-random behavior of HERV elements and families and thus suggest a strong HERV tropism acting within human organs.

In line with this idea, we focused on differential expression between normal and tumor tissues in pairwise analyses. The use of SAM-FDR gold standard statistical tests [80] led to the identification of a variable number of elements that are sensitive to the state of differentiation. We took the responsibility of false-positive results using a high FDR value but we also assumed that, by using a test with low stringency, we did not miss any interesting elements. Testis here again appears to be the most predisposed context to HERV differential activity with more than 1,000 DEP composed of almost two-thirds of tissue-specific probesets. Notably we highlighted a significant number of probesets with strong and specific expression variation between normal and cancerous colon samples. The RT-PCR experiments we set up to validate HERV tropism and differential expression showed that HERV-V2 overall trends are accurate. Nevertheless, discrepancies between microarray and RT-PCR have also been observed, which may reflect a lower sensitivity of the chip as opposed to RT-PCR, e.g. due to the intrinsic sensitivity of the whole transcriptome amplification or to a target-dependent unbalanced amplification. For ovary and lung analysis, although the number of DEP seems impressive, only a few probesets deviate from low values. In addition, we did observe variable levels of genomic DNA contamination within lung samples, which may have biased the result of analysis. Altogether, although promising, the transfer of these results into biomarkers will require further clinical studies based on relevant dedicated procedures [81], notably taking into account inter-individual variations.

### Specialization of Human LTR Function

After a retrovirus has integrated the host genome, its two flanking LTR sequences are strictly identical, yet the alteration of HERV structures and the genetic drift over time may provide a favorable context for both natural and alternative LTR functions. As a result in the current human genome, the estimated 200,000 HERV LTRs can be seen as a wild collection of promoter and polyA elements. Based on this concept, we identified 326 promoter LTRs, 209 polyA LTRs, 25 readthrough LTRs and 672 silent LTRs among the 1513 evaluated LTRs. Confirmation analysis based on HERV-W-associated ESTs revealed that putative splicing events excluding U3 regions occurred in some cases, which may lead to an overestimation of promoter functions. Conversely, we did not assign promoter functions to LTRs lacking probes in U3 but exhibiting high positive signals in U5. In particular, we identified 34% of active HML-2 promoters. This is slightly less than the GREM experimental method that showed at least 50% of HERV-K HML-2 LTR serve as in vivo promoters [62], [36]. Some of the elements identified with GREM were found in our study but it is somewhat disturbing to find only a poor correlation (19%). This could be due to inter individual variations among tissue samples in both studies, as only one testicular parenchyma was used to implement the GREM methology [62]. Alternatively, given that GREM is a PCR-based method, the analysis of transcribed HERV sequences can be more sensitive than with microarrays but conversely can be complicated by recombination events during PCR [58].

Most of the function characterizations concern solo LTRs (398 out of 535; 74%). In detail, we characterized 247 promoter solo LTRs and 151 polyA solo LTRs. If we look at solo LTRs regardless of their family, we are inclined to consider that these structures, originating from recombination phenomena, are more likely to exert promoter rather than polyA functions. However, the relative amount of promoter and polyA solo LTRs varies remarkably from one family to another. Within the HERV-K HML-5 family, we only characterized promoter solo LTRs. The HERV-W, HERV-E 4.1, HERV-FRD and HERV-K HML-2 families similarly showed a predominant set of solo LTRs with promoter functions. It is noteworthy that among the 6 HERV families we studied, the oldest, HERV-H, gives the most significant example of polyA solo LTR overrepresentation. The observed biases in solo LTR specialization may result from an intrinsic property of the natural history of each family, as exemplified in a different context by the LINE-1-mediated spreading of a significant proportion of the HERV-W family [82]. Alternatively we cannot exclude an orientated and irreversible genetic drift within LTR sequences. Further functional comparative analysis of evolutionary-conserved solo LTRs may permit to address these hypotheses.

We also examined the 5′ end of the 45 promoter 3′LTR elements. The proportion of 5′-truncated structures in this subset is not higher compared to other HERV proviruses. However, when a function can be attributed, the existing 5′LTR is silent. This observation can suggest a loss of fixation of transcription factors. Indeed, different works on proto-oncogene activation induced by retrovirus insertion have showed that the 3′LTR can initiate alternative transcription of cellular genes only if the insertion was accompanied by an inactivation of the 5′LTR of the provirus [83], a concept referred to as promoter occlusion [84]. Thus, the description of 45 promoter 3′LTRs in this study appears consistent with the concept of promoter occlusion.

Astonishingly, promoter and polyA lists have no LTR in common, a strong trend we called operational determinism. This was observed using both the 79 normal et cancerous tissue panel and the 6 cell lines. Thus, despite environmental changes over time, active LTRs seem to feature unique specialized functions. Nevertheless, HERV-W-associated ESTs showed that in some contexts, only a readtrough phenomenon can replace or be added to promoter or polyA function. This finding is compatible with operational determinism but suggests the presence of weak promoter or polyA activities. In addition, attempts to validate LTR functions by leveraging EST data have faced the possibility of alternative transcription initiations. Indeed, alternative initiation sites have been proposed for the promoter of ERVWE1 following mung bean nuclease protection assays [65]. These two alternative sites are located 71 bp and 75 bp upstream from the site we defined by RACE as the R border [63], [61], respectively. Moreover, due to genetic drift, the location of initiation sites within HERV LTRs may be more flexible than for exogenous retroviruses.

### HERV Functions and Genomic Environment

Gene density in the environment of active promoter LTRs is significantly higher than for silent LTRs as previously observed for the HML-2 family [36]. Notably, this behavior was also shared by LTRs exhibiting polyA function. Such observations could be interpreted in two ways: either chromosomal regions with high transcriptional activity promote HERV activity as a side effect (e.g.: bringing transcription factors together with DNA strand opening), or there is a functional contribution of active LTRs to human gene regulation in a way that would be of benefit to the genome. Conversely, exclusion of methylated silent LTRs from gene-rich regions preclude methylation spreading and then silencing of conventional genes as previously suggested for transposable elements [85]. The set of 99 intronic LTR elements investigated here presented a 3.7 fold bias in favor of antisense-oriented insertion, similar to the 2 to 4.5 range previously described [86], [87]. As previously proposed, this suggests a strong selection against LTR elements in the sense direction and consequently argues that LTRs found in the same transcriptional orientation are much more likely to have a detrimental effect [87]. It is noteworthy that the antisense orientation bias appears similar for silent and transcriptionally active LTRs. Regarding surrounding genes, this may reflect an overall weak transcriptional activity as observed for a set of proviruses and solo LTRs belonging to the HERV-W family [88]. Alternatively this could represent substantial and therefore gene-independent transcription events in altered cellular contexts.

Among the 1133 intergenic LTR elements, 288 (25%) were promoter LTRs, 184 (16%) polyA LTRs and 639 (56%) silent LTRs. Comparison of the gene environment of those intergenic LTRs highlighted two points. Unexpectedly, an approximate 8 kb interval upstream of intergenic promoter LTRs was characterized by a drastic under-representation of sense genes. This result was considered relevant due to the significant number of LTRs (n = 228) and the absence of LTR-associated multigene families which may skew the results. This suggests that a sense-intergenic promoter LTR can only survive at a certain distance of a sense gene, otherwise it would have a detrimental effect on the gene. Such a location may contribute to the usage of acceptor donor sites together with alternative polyA signal which may alter the original transcript as proposed for intronic elements [87]. Second, a mirror situation consisting in an 8 kb window was observed upstream from silent LTRs, showing a decrease in antisense genes compared to sense genes. Although no obvious explanation can be provided to date, it is striking to note that such a symmetrical 8 kb region was recently shown to correspond to the maxima of LTR density around transcription start site of tissue-specific genes [89].

### Conclusion

This microarray-based approach unveiled the expression of 1,718 distinct HERV loci and identified 326 promoter LTRs and 209 polyA LTRs in a broad range of tissues. Further systematic quantitative analysis is required to gain insight on the relative variation of expression of HERV sequences and their adjacent cellular genes. In particular, looking at different stages of cell differentiation may accelerate the identification of alternative promoters as already documented for a subset of genes in the mouse embryo [90].

In addition to the preservation of transcription factor binding sites, two important features determining the control of HERV expression consist of the LTR methylation status [91], [92], [93], [94], [61], [95] and the chromatin context associated to post-translational histone modifications [95]. Locus-specific LTR hypomethylation was observed both during placental development [91], [92], [93] and in testis and colon cancers [94], [61], [95]. Thus, such whole transcriptome approach together with LTR function identification and further characterization of associated epigenetic marks may help to discriminate between *statu quo*, conflict and cooperation, the components of a many-facetted relationship between retrotransposons and their metazoan hosts.

## Materials and Methods

### Chip Design

#### HERV database

A database for genomic HERV elements was constructed following a 4-step process: (i) for each HERV family, we defined a prototype by choosing the most representative and complete HERV element present in the human genome. (ii) Functional U3/U5/gag/pol/env parts were labeled on the prototypes. (iii) These sequences were then used as an input reference library for RepeatMasker [96] (see the details of the prototypes in Table S1). The search for HERV functional sequences was extended to the entire human genome (NCBI 36/hg18) allowing a maximum 20% divergence with prototype sequences. (iv) The functional sequences identified were lastly assembled into annotated HERV elements and were implemented in an owner database, so-called HERVgDB3. HERVgDB3 contains 10,035 distinct HERV elements belonging to 6 HERV families, including complete and partial proviruses (Table 1*a*).

### Probeset Design

The probe design steps aimed ultimately to define probesets for the functional parts of each HERV element that belongs to HERVgDB3. We first generated all possible and overlapping 25-mer tracks for any given HERV sequence of HERVgDB3, leading to an initial pool of candidate probes. We then evaluated the cross-hybridization risk of each candidate probe using local alignment versus the entire human genome (NCBI 36/hg18) as a model of hybridization, supported by an internally developed alignment scoring function called EDA+. The EDA+ principle is based on instability induced by any mismatch within the hybridization between probe and target. Using EDA+, the impact of mismatches is cumulative and modulated regarding their type, their position and the size of the interval between two mismatches. A threshold on the cumulative weight is then defined to consider the hybridization as probable or not. Note that no specific thermodynamic parameter was added to the model. The relevance of this score was evaluated independently (data not shown). EDA+ was applied to any local alignment between a candidate probe and the human genome, computed using the KASH algorithm [97]. Probes that meet the alignment-EDA+ criteria were definitively selected to enter the design process. This last step finally grouped the selected probes in order to constitute probesets for any given functional part of the HERV elements collection. When more than 10 probes can be used to create a probeset, we make a selection to obtain a homogeneous distribution of probes along the functional part.

### Custom HERV GeneChip Microarray

The custom HERV GeneChip integrates 23,583 HERV probesets (88,592 probes) and can discriminate 5,573 distinct HERV elements, composed of complete and partial proviruses (Table 1*b*). In addition to the HERV repertoire, a set of mismatch declinations (37,200 probes), initially based on 19 perfect match (PM) probesets belonging to the commercial Affymetrix HG_U133_PLUS2 chip, serves to evaluate and improve the EDA+ hybridization scoring function (data not shown). The standard Affymetrix control probes for unbiased amplification and hybridization were also included in the microarray.

### Sample Description

#### Tissue samples and cell lines

Matched-pair tumor/normal RNA samples of colon (3), breast (8), ovary (3), uterus (3) and prostate (1) were purchased from Clinisciences. Additional First Choice human tumor/normal RNA samples of colon (1), ovary (1), uterus (1), testis (1), lung (1) and prostate (2), plus normal placenta sample (1), were obtained from Life Technologies. The Centre de Ressources Biologiques of Nancy provided epidermoid carcinoma and normal adjacent lung RNA samples (9) and the Centre Hospitalier Lyon-Sud performed macro dissections on radical prostatectomy specimens (5) to isolate cancer tissue from normal tissue. Details on samples are provided in Table S2.

The human prostate epithelial cell line RWPE1 and the chemically stressed-derived WPE1-NA22, WPE1-NB14, WPE1-NB11, WPE1-NB26 [98] as well as the v-Ki-Ras-transformed RWPE2 [99] cell lines were obtained from the CelluloNet of the UMS3444/US8 BioSciences Gerland Lyon-Sud.

#### Ethical considerations

The human tissue specimens provided by the Centre Hospitalier Lyon-Sud and by the Centre de Ressources Biologiques of Nancy were obtained in compliance with the ICH-GCP regulations, current European and French legislations. A ‘non-interventional’ biomedical research protocol for tissue samples conservation after a prostate surgery has been set-up at the Centre Hospitalier Lyon-Sud with the approval of the Ethics Committee in Lyon (CPP Sud-Est 2). Therefore, patients admitted to the urology department in the Centre Hospitalier Lyon-Sud were informed and gave voluntary, signed informed consent prior to any tissue sample conservation and for research use. Patients admitted to the Nancy Hospital were informed that their sample tissue after the lung surgery will be conserved at the Centre de Ressources Biologiques de Nancy for research use according to the French bioethics law (2004). Clinisciences and Life Technologies signed an agreement to ensure that the tissue samples were obtained in compliance with ICH-GCP standards.

### Molecular Biology Analysis

#### RNA extraction

RNA was extracted from macro-dissected radical prostatectomies following the Trizol protocol (Invitrogen) and was purified on Rneasy columns (Qiagen). The quality of all RNA samples was assessed with the Bioanalyser 2100 capillary electrophoresis device using the RNA Nano Chips kit (Agilent).

#### Target amplification, labeling and microarray hybridization

cDNA synthesis and amplification were performed using 50 ng of RNA, using the WT-Ovation RNA Amplification System kit (Nugen). Briefly, amplification was initiated both at the 3′ end and randomly throughout the whole transcriptome, and this was followed by reverse transcriptase/RNAse H mix step before SPIA linear and single strand amplification. Amplified ssDNA products were purified using the QIAquick purification kit (Qiagen), total DNA concentration was measured using the NanoDrop 1000 spectrophotometer (Termo Scientific) and the product quality was checked on the Bioanalyser 2100. Two micrograms of purified ssDNA were fragmented into 50–200 bp fragments by DNAseI treatment and were 3′-labeled using a terminal transferase recombinant kit (Roche). The resulting target was mixed with standard hybridization controls and B2 oligonucleotides following the recommendations of the supplier. The hybridization cocktail was heat-denatured at 95°C for 2 minutes, incubated at 50°C for 5 minutes and centrifuged at 16,000 g for 5 minutes to pellet the residual salts. The HERV GeneChip microarrays were prehybridized with 200 µl of hybridization buffer and placed under stirring (60 rpm) in an oven at 50°C for 10 minutes. The hybridization buffer was then replaced by the denatured hybridization cocktail. Hybridization was performed at 50°C for 18 hours in the oven under constant stirring (60 rpm). Washing and staining were carried out according to the protocol supplied by the manufacturer, using a fluidic station (GeneChip fluidic station 450, Affymetrix). The arrays were scanned using a fluorometric scanner (GeneChip scanner GS 3000, Affymetrix).

#### Real-time PCR

A set of locus-specific PCR primers was designed using Primer3 and the NCBI Primer-BLAST software and then checked *in silico* at UCSC (http://genome.ucsc.edu). Primers were ordered from Eurogentec. For each individual PCR system, a range of amplifications, followed by High Resolution Melting (HRM) analysis and product sequencing, was performed on genomic DNA to control the specificity of the products and to determine optimal experimental melting temperature (Tm). For each tissue, individual samples were pooled in order to compare results from RT-PCR with the data from microarrays. 50 ng of total RNA of each sample were DNAse-treated and reverse-transcribed using the QuantiTec Reverse Transcription Kit (Qiagen). Reverse-transcriptase-free reactions were carried out to verify the absence of contaminating genomic DNA. SYBR green experiments were set up using the Type-it HRM PCR kit (Qiagen) in 10 µL final reaction volume with 5 µM primers and a 20-fold dilution of the cDNA. PCR amplifications were carried out in Rotor-disc 100 wrapped discs devised for the Rotor Gene Q (Qiagen). Housekeeping genes G6PD, GAPDH and HPRT were analyzed in the same experiment as the target transcripts. Amplifications of cDNA were performed as follows: a 5-min denaturation step at 95°C, followed by 45 cycles (95°C for 10 s, Tm for 30 s, 72°C for 10 s) and HRM analysis (from 65°C to 95°C, 0.1°C increments every 2 s) to control the product purity. Each reaction was performed in duplicate. The second derivative method was used to assess the amplification efficiency (Eff). Relative expression (RLE) for each system is Eff^ΔCt^ (ΔCt = Ct_min_–Ct_sample_). All data were normalized by the geometric mean of the RLE of the three housekeeping genes.

### Bioinformatics

#### Chip analysis

The quality control (QC) of the microarrays was assessed using the standard Affymetrix controls to verify that the chips met the criteria. In addition, the dataset was explored to highlight unexpected batch effects and to correct them before statistical analysis. The distributions of intensities for probes and probesets were plotted to test different putative covariate effects (e.g.: dates of amplification and hybridization, people in charge of the experiment, lots of reagents). The following representations were used: the log intensity value distribution (density plots and box plots), the median absolute deviation (MAD) versus the intensity median (MAD-Med plots), the background plots and nuse plots and finally the relative log expression (RLE) plots. A strong batch effect related to the experimental operator was identified, as well as residual batch effects related to the amplification dates within each operator. A customized pre-processing strategy was thus selected to correct these technical and undesired effects.

The data pre-processing included a background correction based on the tryptophan probe baseline signal, followed by normalization and summarization steps involving a double batch effect correction. In brief, the background of each chip was estimated as the 15^th^ percentile of the intensity values of the tryptophan probes, then the robust microarray averaging (RMA) process [100] was applied within each operator batch using the configuration of quantile normalization followed by median polish summarization. This process was applied independently for each amplification date batch. After that, a two-step combining batches (COMBAT) method [101] was applied, first within each operator dataset in order to merge the date effects of a given operator, and second within the entire chips set in order to merge the operator datasets together (see Figure S1). The COMBAT method constructs a model for each gene, formally written as:
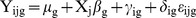

*Y_ijg_* is the signal measured for the gene *g* when the sample *j* is processed in the batch *I*; *µ_g_* is a mean expression level for the gene *g*; we considered a single biological covariate *X* (here a qualitative variable including the origin and the state of the tissue); *β_g_* defines the level of differential expression related to biological categories (the parameter we are looking for); parameters *γ_ig_* and *δ_ig_* are the additive and multiplicative error components that define the batch *i* (they are gene-specific) and *ε_ijg_* is the error that follows N(*0*, *σ_g_*).

After all the chips were normalized, expression values of individual chips were grouped into sets of samples as described in Table S2. If no precision is given, all the results illustrated and discussed in this study are based on the values of the sets of samples.

Partitioning clustering was applied to the normalized expression values using a Euclidean distance function algorithm to determine similarities between observations. The final number of clusters was decided after iterative corrections combining algorithm auto-decisions and fine adjustments through direct observations of the resulting dataset arrangement. The minimum number of probesets required to form an expression cluster was empirically set at 6.

The search for differentially expressed genes (DEG) implied a classical significant analysis of microarray (SAM) procedure [80] followed by a false discovery rate (FDR) correction [102]. The dataset was filtered to exclude the probesets for which expression values were less than 2^6^ in all tissues. A FDR cut-off of 20% was applied.

#### Genomic environment

Homemade perl scripts were developed to request and extract information from the human genome build NCBI 36/hg18 and the RefGene annotation table (UCSC). Gene density and %GC content calculation were evaluated by default in the +/−50 kb surrounding environment, starting from the HERV element ends.

#### Expressed Sequenced Tag (EST) analysis

The blastn algorithm (NCBI blastn v.2.2.25) was used to compare HERV sequences to EST libraries. A cut-off of 97% was retained as a compromise between the extreme similarity existing between loci of the same family and the polymorphism in the human population, ranging from 1 out of 0.31 kb in repeats to 1 out of 1.8–2.0 kb in coding regions (Nickerson, 1998}. If no precision is given, the default parameters used for alignment were: alignment length >200 bp; EST/sequence alignment coverage >85%.

#### Software and data

QC, pre-processing and DEG analysis were performed using R statistical software [103], packages from the Bioconductor project [104] and homemade R packages. The clustering algorithm used for this study is implemented in Partek Genomics Suite 6.5. Geneious 5.0 was used for primer design and EST analysis. The complete experimental set comprises 113 microarrays. Affymetrix data files (.cel) are available upon request.

## Supporting Information

Figure S1 **Effect of RMA-COMBAT normalization.** Distribution of intensities within the dataset before (upper part) and after (lower part) RMA-COMBAT normalization. Each boxplot represents a single chip and the colors refer to experimental batches.
(PDF)

Figure S2 **Correlations between microarray and RT-PCR results.** Normalized values of microarray and RT-PCR experiments are given for 12 independent HERV sequences that belong to 8 distinct HERV loci. Correlations close to 1 indicate a strong positive linear relationship and therefore confirm the findings. Correl  =  Cov_microarray;RT-PCR/_(sd_microarray_*sd_RT-PCR_).
(PDF)

Figure S3 **RT-PCR analyses of LTR promoter functions.** The promoter activity of 3 independent LTRs was evaluated in RT-PCR. Relative expression of U5 *vs* U3 is given by Fc_U5/U3_ =  (Eff_U3_^CtU3^)/(Eff_U5_^CtU5^). Values greater than 1 indicate a promoter activity. An asterisk (*) highlights tissues for which the promoter activity has been unequivocally found using the microarrays. In the particular case of 1100414_2 no probeset was defined within the LTR and consequently the promoter activity in testicular tumor could not be detected using microarrays.
(PDF)

Figure S4 **Pictures illustrating alignments of HERV-W loci with their best EST counterpart.** Each alignment is designated by the name of the locus as it stands on the microarray, followed by the name of the most similar EST. The alignment explicitly states the retroviral structure including LTR U3, R and U5 subdomains, as well as flanking regions. Probes defined on the array are indicated by grey arrows. The sequence used for the query is represented as well as the EST retained for analysis, as developed in Table S7. Accession number and EST count are shown. Arbitrary blue numbering of HERV subdomain and the aligned EST together with blue vertical bars are indicated when required to facilitate the reading, e.g. clones overlapping U5 and 5′ flanking region for 400207_w-AI738459.jpg. Best score EST aligned with 5′ (700341_w-ERVWE1_5LTR.jpg) and 3′ (700341_w-ERVWE1_3LTR.jpg) LTRs of the ERVWE1 locus are included to highlight the limits of information provided by ESTs.
(PDF)

Table S1 **HERV prototypes used for the construction of HERV-gDB3.** Accession numbers, genomic localizations and the limits of the functional region within the prototype sequences (U3, R, U5, gag, pol env) are given for the 6 HERV families studied. *^a^* for HERV identification and gene cutting out. *^b^* for LTR sub region cutting out.
(PDF)

Table S2 **Biological samples included in the study.** List of biological samples included in the study (samples) and used in the composition of analysis groups (set of samples). Information on pathological status, age and sex are provided when available. Matched tumoral/normal samples are indicated (paired with). An asterisk (*) highlights samples that were not used for the microarray study.
(PDF)

Table S3 **Genomic coordinates of active and functional HERV sequences.** Genomic coordinates refer to the human genome version NCBI 36/hg18. Each HERV locus is designated by a single identifier (locus id). The table summarizes the different observations mentioned in the study, i.e. whether the locus shows expression patterns (tropism), is differentially expressed between normal and cancer samples (DGE) or exhibits functional LTRs (LTR functions). Two ‘x’ in the “LTR functions” box associated with one locus reflect distinct functions for each LTR of the same provirus.
(XLS)

Table S4 **Identification of HML-2 repetitive elements characterized by independent methods.** From left to right the genomic location (NCBI 36/hg18), the individual HML-2 locus sequence name, the tropism of expression deduced from the microarrays, the differential expression and the LTR functions as depicted in Table S3, the references from which data were obtained taking into consideration either EST analysis [59], genomic repeat expression monitoring (GREM) for experimental genome-wide identification of promoter-active repetitive elements [62], PCR-sequencing [48] or array-based approach [61] are given. The original designation of the HERV loci is given for each study. We added April 2012 EST query information obtained using the method developed in Table S7. Statistics concerning this analysis are given at the bottom of the table and include, for each study, the number of elements, the number of shared elements, the number of active elements and the correlation between our work and each individual study.
(XLS)

Table S5 **Matching of tissue-specific HERV sequences with Expressed Sequenced Tag (EST) databases.** The CleanEST database [105] was used to retrieve ESTs associated with tissues of interest in order to construct 6 reference EST groups: colon (311122 ESTs), lung (441913 ESTs), ovary (123944 ESTs), placenta (321881 ESTs), prostate (69860 ESTs) and testis (264243 ESTs). Each EST group was blasted against the HERV sequences composing the expression profiles shown in Figure 1, following the procedure detailed in the EST analysis part of the materials and methods section. Hits were normalized by the total number of HERV loci of the expression profile and by the total number of ESTs forming the reference group. The ranking of the value is associated with a color code highlighting the enrichment of tissue-associated ESTs: green (1/6), yellow (2/6) and red (>2/6).
(PDF)

Table S6 **Primers used for RT-PCR experiments.** Forward and reverse primer sequences used for RT-PCR analyses. The Tm of each primer pair was determined as described in the related materials and methods section. The domain of application is indicated (normalization, tropism, promoter function).
(PDF)

Table S7 **Identification of Expressed Sequenced Tags (ESTs) putatively associated with active HERV-W repetitive elements.** We used Megablast to compare HERV sequences to EST libraries using Geneious 5.0 software and NCBI libraries. A cut-off of 97% was retained as a compromise between the extreme similarity existing between loci of the same family and the polymorphism in the human population ranging from 1 out of 0.31 kb in repeats to 1 out of 1.8–2.0 kb in coding regions [106]. From left to right, the genomic location (NCBI 36/hg18), the individual HERV-W locus sequence name, the tropism of expression deduced from the microarrays, the differential expression and the LTR functions as depicted in the Table S3, the LTR associated structure (i.e: provirus, solo LTR, partial provirus with either 5′ or 3′ LTR), the EST scores, the reference accession numbers of the ESTs, the EST length in bp, the EST coverage of the LTR query (i.e: 100% or numbering when <100%), the LTR-element covered regions (i.e: U3, R, U5, gag, pol, env, 5′ or 3′ flanking region), the information concerning the additional coverage of clones and the existence of additional clones in flanking regions, the previous identification and designation of the locus, the EST-associated proposed function, and the name of the pictures illustrating alignments of HERV loci with their best EST counterpart as detailed in Figure S4 are given. Parameters used for Megablast query with Geneious 5.0 are indicated at the bottom of the table, as well as statistics concerning the query and a color code highlighting the correlation between array and EST LTR-deduced functions.
(XLS)

## References

[1] Boller K, Frank H, Lower J, Lower R, Kurth R (1983). Structural organization of unique retrovirus-like particles budding from human teratocarcinoma cell lines.. J Gen Virol 64 (Pt.

[2] Boller K, Konig H, Sauter M, Mueller-Lantzsch N, Lower R (1993). Evidence that HERV-K is the endogenous retrovirus sequence that codes for the human teratocarcinoma-derived retrovirus HTDV.. Virology.

[3] Lyden TW, Johnson PM, Mwenda JM, Rote NS (1994). Ultrastructural characterization of endogenous retroviral particles isolated from normal human placentas.. Biol Reprod.

[4] Smit AFA (1999). Interspersed repeats and other mementos of transposable elements in mammalian genomes.. Curr Opin Genet Dev.

[5] Lander ES, Linton LM, Birren B, Nusbaum C, Zody MC (2001). Initial sequencing and analysis of the human genome.. Nature.

[6] Venter JC, Adams MD, Myers EW, Li PW, Mural RJ (2001). The sequence of the human genome.. Science (New York, N Y ).

[7] Mouse Genome Sequencing Consortium (2002). Initial sequencing and comparative analysis of the mouse genome.. Nature.

[8] Bannert N, Kurth R (2004). Retroelements and the human genome: new perspectives on an old relation.. Proc Natl Acad Sci U S A.

[9] Bannert N, Kurth R (2006). The evolutionary dynamics of human endogenous retroviral families.. Annu Rev Genomics Hum Genet.

[10] Katzourakis A, Tristem M, Sverdlov E (2004). Phylogeny of Human Endogenous and Exogenous Retroviruses..

[11] Pérot P, Montgiraud C, Lavillette D, Mallet F, Larsson LI (2011). A Comparative Portrait of Retroviral Fusogens and Syncytins..

[12] Bjerregaard B, Holck S, Christensen IJ, Larsson LI (2006). Syncytin is involved in breast cancer-endothelial cell fusions.. Cell Mol Life Sci.

[13] Strick R, Ackermann S, Langbein M, Swiatek J, Schubert SW (2007). Proliferation and cell-cell fusion of endometrial carcinoma are induced by the human endogenous retroviral Syncytin-1 and regulated by TGF-beta.. J Mol Med.

[14] Sun Y, Ouyang DY, Pang W, Tu YQ, Li YY (2010). Expression of syncytin in leukemia and lymphoma cells.. Leukemia research.

[15] Boese A, Sauter M, Galli U, Best B, Herbst H (2000). Human endogenous retrovirus protein cORF supports cell transformation and associates with the promyelocytic leukemia zinc finger protein.. Oncogene.

[16] Armbruester V, Sauter M, Roemer K, Best B, Hahn S (2004). Np9 protein of human endogenous retrovirus K interacts with ligand of numb protein X. J Virol.

[17] Denne M, Sauter M, Armbruester V, Licht JD, Roemer K (2007). Physical and functional interactions of human endogenous retrovirus proteins Np9 and rec with the promyelocytic leukemia zinc finger protein.. J Virol.

[18] Kaufmann S, Sauter M, Schmitt M, Baumert B, Best B (2010). Human endogenous retrovirus protein Rec interacts with the testicular zinc-finger protein and androgen receptor.. The Journal of general virology.

[19] Sauter M, Schommer S, Kremmer E, Remberger K, Dolken G (1995). Human endogenous retrovirus K10: expression of Gag protein and detection of antibodies in patients with seminomas.. J Virol.

[20] Boller K, Janssen O, Schuldes H, Tonjes RR, Kurth R (1997). Characterization of the antibody response specific for the human endogenous retrovirus HTDV/HERV-K.. J Virol.

[21] Goedert JJ, Sauter ME, Jacobson LP, Vessella RL, Hilgartner MW (1999). High prevalence of antibodies against HERV-K10 in patients with testicular cancer but not with AIDS.. Cancer Epidemiol Biomarkers Prev.

[22] Rearden A, Magnet A, Kudo S, Fukuda M (1993). Glycophorin B and glycophorin E genes arose from the glycophorin A ancestral gene via two duplications during primate evolution.. J Biol Chem.

[23] Schwartz A, Chan DC, Brown LG, Alagappan R, Pettay D (1998). Reconstructing hominid Y evolution: X-homologous block, created by X-Y transposition, was disrupted by Yp inversion through LINE-LINE recombination.. Hum Mol Genet.

[24] Long M (2001). Evolution of novel genes.. Curr Opin Genet Dev.

[25] Johnson WE, Coffin JM (1999). Constructing primate phylogenies from ancient retrovirus sequences.. Proc Natl Acad Sci U S A.

[26] Hughes JF, Coffin JM (2001). Evidence for genomic rearrangements mediated by human endogenous retroviruses during primate evolution.. Nat Genet.

[27] Hughes JF, Coffin JM (2005). Human endogenous retroviral elements as indicators of ectopic recombination events in the primate genome.. Genetics.

[28] Paces J, Pavlicek A, Paces V (2002). HERVd: database of human endogenous retroviruses.. Nucleic Acids Res.

[29] Belshaw R, Watson J, Katzourakis A, Howe A, Woolven-Allen J (2007). Rate of recombinational deletion among human endogenous retroviruses.. J Virol.

[30] Brosius J (1999). Genomes were forged by massive bombardments with retroelements and retrosequences.. Genetica.

[31] Jern PCoffin JM (2008). Effects of retroviruses on host genome function.. Annual review of genetics.

[32] Samuelson LC, Wiebauer K, Gumucio DL, Meisler MH (1988). Expression of the human amylase genes: recent origin of a salivary amylase promoter from an actin pseudogene.. Nucleic Acids Res.

[33] Medstrand P, Landry JR, Mager DL (2001). Long terminal repeats are used as alternative promoters for the endothelin B receptor and apolipoprotein C-I genes in humans.. J Biol Chem.

[34] Dunn CA, Medstrand P, Mager DL (2003). An endogenous retroviral long terminal repeat is the dominant promoter for human beta1,3-galactosyltransferase 5 in the colon.. Proc Natl Acad Sci U S A.

[35] van de Lagemaat LN, Landry JR, Mager DL, Medstrand P (2003). Transposable elements in mammals promote regulatory variation and diversification of genes with specialized functions.. Trends Genet.

[36] Buzdin A, Kovalskaya-Alexandrova E, Gogvadze E, Sverdlov E (2006). At least 50% of human-specific HERV-K (HML-2) long terminal repeats serve in vivo as active promoters for host nonrepetitive DNA transcription.. J Virol.

[37] Romanish MT, Lock WM, van de Lagemaat LN, Dunn CA, Mager DL (2007). Repeated recruitment of LTR retrotransposons as promoters by the anti-apoptotic locus NAIP during mammalian evolution.. PLoS Genet.

[38] Conley AB, Piriyapongsa J, Jordan IK (2008). Retroviral promoters in the human genome.. Bioinformatics.

[39] Dunn CA, Romanish MT, Gutierrez LE, van de Lagemaat LN, Mager DL (2006). Transcription of two human genes from a bidirectional endogenous retrovirus promoter.. Gene.

[40] Faulkner GJ, Kimura Y, Daub CO, Wani S, Plessy C (2009). The regulated retrotransposon transcriptome of mammalian cells.. Nat Genet.

[41] Ruda VM, Akopov SB, Trubetskoy DO, Manuylov NL, Vetchinova AS (2004). Tissue specificity of enhancer and promoter activities of a HERV-K(HML-2) LTR.. Virus Res.

[42] Prudhomme S, Oriol G, Mallet F (2004). A retroviral promoter and a cellular enhancer define a bipartite element which controls env ERVWE1 placental expression.. J Virol.

[43] Mager DL, Hunter DG, Schertzer M, Freeman JD (1999). Endogenous retroviruses provide the primary polyadenylation signal for two new human genes (HHLA2 and HHLA3).. Genomics.

[44] Gogvadze E, Stukacheva E, Buzdin A, Sverdlov E (2009). Human-specific modulation of transcriptional activity provided by endogenous retroviral insertions.. J Virol.

[45] Cohen CJ, Lock WM, Mager DL (2009). Endogenous retroviral LTRs as promoters for human genes: a critical assessment.. Gene.

[46] Cooper SJ, Trinklein ND, Anton ED, Nguyen L, Myers RM (2006). Comprehensive analysis of transcriptional promoter structure and function in 1% of the human genome.. Genome research.

[47] Kimura K, Wakamatsu A, Suzuki Y, Ota T, Nishikawa T (2006). Diversification of transcriptional modulation: large-scale identification and characterization of putative alternative promoters of human genes.. Genome research.

[48] Flockerzi A, Ruggieri A, Frank O, Sauter M, Maldener E (2008). Expression patterns of transcribed human endogenous retrovirus HERV-K(HML-2) loci in human tissues and the need for a HERV Transcriptome Project.. BMC Genomics.

[49] de Parseval N, Lazar V, Casella JF, Benit L, Heidmann T (2003). Survey of human genes of retroviral origin: identification and transcriptome of the genes with coding capacity for complete envelope proteins.. J Virol.

[50] Wang-Johanning F, Frost AR, Jian B, Azerou R, Lu DW (2003). Detecting the expression of human endogenous retrovirus E envelope transcripts in human prostate adenocarcinoma.. Cancer.

[51] Smallwood A, Papageorghiou A, Nicolaides K, Alley MK, Jim A (2003). Temporal regulation of the expression of syncytin (HERV-W), maternally imprinted PEG10, and SGCE in human placenta.. Biol Reprod.

[52] Okahara G, Matsubara S, Oda T, Sugimoto J, Jinno Y (2004). Expression analyses of human endogenous retroviruses (HERVs): tissue-specific and developmental stage-dependent expression of HERVs.. Genomics.

[53] Buscher K, Trefzer U, Hofmann M, Sterry W, Kurth R (2005). Expression of human endogenous retrovirus K in melanomas and melanoma cell lines.. Cancer Res.

[54] Seifarth W, Frank O, Zeilfelder U, Spiess B, Greenwood AD (2005). Comprehensive analysis of human endogenous retrovirus transcriptional activity in human tissues with a retrovirus-specific microarray.. J Virol.

[55] Forsman A, Yun Z, Hu L, Uzhameckis D, Jern P (2005). Development of broadly targeted human endogenous gammaretroviral pol-based real time PCRs Quantitation of RNA expression in human tissues.. J Virol Methods.

[56] Muradrasoli S, Forsman A, Hu L, Blikstad V, Blomberg J (2006). Development of real-time PCRs for detection and quantitation of human MMTV-like (HML) sequences HML expression in human tissues.. Journal of virological methods.

[57] Pichon JP, Bonnaud B, Mallet F (2006). Quantitative multiplex degenerate PCR for human endogenous retrovirus expression profiling.. Nat Protoc.

[58] Flockerzi A, Maydt J, Frank O, Ruggieri A, Maldener E (2007). Expression pattern analysis of transcribed HERV sequences is complicated by ex vivo recombination.. Retrovirology.

[59] Stauffer Y, Theiler G, Sperisen P, Lebedev Y, Jongeneel CV (2004). Digital expression profiles of human endogenous retroviral families in normal and cancerous tissues.. Cancer immunity : a journal of the Academy of Cancer Immunology.

[60] Oja M, Peltonen J, Blomberg J, Kaski S (2007). Methods for estimating human endogenous retrovirus activities from EST databases.. BMC Bioinformatics.

[61] Gimenez J, Montgiraud C, Pichon JP, Bonnaud B, Arsac M (2010). Custom human endogenous retroviruses dedicated microarray identifies self-induced HERV-W family elements reactivated in testicular cancer upon methylation control.. Nucleic Acids Res.

[62] Buzdin A, Kovalskaya-Alexandrova E, Gogvadze E, Sverdlov E (2006). GREM, a technique for genome-wide isolation and quantitative analysis of promoter active repeats.. Nucleic Acids Res.

[63] Blond JL, Beseme F, Duret L, Bouton O, Bedin F (1999). Molecular characterization and placental expression of HERV-W, a new human endogenous retrovirus family.. J Virol.

[64] Prudhomme S, Oriol G, Mallet F (2004). A retroviral promoter and a cellular enhancer define a bipartite element which controls env ERVWE1 placental expression.. J Virol.

[65] Cheng YH, Richardson BD, Hubert MA, Handwerger S (2004). Isolation and characterization of the human syncytin gene promoter.. Biol Reprod.

[66] Mallet F, Bouton O, Prudhomme S, Cheynet V, Oriol G (2004). The endogenous retroviral locus ERVWE1 is a bona fide gene involved in hominoid placental physiology.. Proc Natl Acad Sci U S A.

[67] Doolittle WF, Sapienza C (1980). Selfish genes, the phenotype paradigm and genome evolution.. Nature.

[68] Ehlhardt S, Seifert M, Schneider J, Ojak A, Zang KD (2006). Human endogenous retrovirus HERV-K(HML-2) Rec expression and transcriptional activities in normal and rheumatoid arthritis synovia.. J Rheumatol.

[69] Birney E, Stamatoyannopoulos JA, Dutta A, Guigo R, Gingeras TR (2007). Identification and analysis of functional elements in 1% of the human genome by the ENCODE pilot project.. Nature.

[70] Hu C, Saenz DT, Fadel HJ, Walker W, Peretz M (2010). The HIV-1 central polypurine tract functions as a second line of defense against APOBEC3G/F.. Journal of Virology.

[71] Mi S, Lee X, Li X, Veldman GM, Finnerty H (2000). Syncytin is a captive retroviral envelope protein involved in human placental morphogenesis.. Nature.

[72] Blaise S, de Parseval N, Benit L, Heidmann T (2003). Genomewide screening for fusogenic human endogenous retrovirus envelopes identifies syncytin 2, a gene conserved on primate evolution.. Proc Natl Acad Sci U S A.

[73] Blaise S, de PN, Heidmann T (2005). Functional characterization of two newly identified Human Endogenous Retrovirus coding envelope genes.. Retrovirology.

[74] Lower R, Lower J, Tondera-Koch C, Kurth R (1993). A general method for the identification of transcribed retrovirus sequences (R-U5 PCR) reveals the expression of the human endogenous retrovirus loci HERV-H and HERV-K in teratocarcinoma cells.. Virology.

[75] Lower R, Tonjes RR, Korbmacher C, Kurth R, Lower J (1995). Identification of a Rev-related protein by analysis of spliced transcripts of the human endogenous retroviruses HTDV/HERV-K.. J Virol.

[76] Armbruester V, Sauter M, Krautkraemer E, Meese E, Kleiman A (2002). A novel gene from the human endogenous retrovirus K expressed in transformed cells.. Clin Cancer Res.

[77] Liang Q, Ding J, Xu R, Xu Z, Zheng S (2009). Identification of a novel human endogenous retrovirus and promoter activity of its 5′ U3.. Biochem Biophys Res Commun.

[78] Pichon JP, Bonnaud B, Cleuziat P, Mallet F (2006). Multiplex degenerate PCR coupled with an oligo sorbent array for human endogenous retrovirus expression profiling.. Nucleic Acids Res.

[79] Liang Q, Xu Z, Xu R, Wu L, Zheng S (2012). Expression Patterns of Non-Coding Spliced Transcripts from Human Endogenous Retrovirus HERV-H Elements in Colon Cancer.. PLoS ONE.

[80] Tusher VG, Tibshirani R, Chu G (2001). Significance analysis of microarrays applied to the ionizing radiation response.. Proceedings of the National Academy of Sciences of the United States of America.

[81] Ptolemy AS, Rifai N (2010). What is a biomarker? Research investments and lack of clinical integration necessitate a review of biomarker terminology and validation schema.. Scandinavian journal of clinical and laboratory investigation Supplementum.

[82] Costas J (2002). Characterization of the intragenomic spread of the human endogenous retrovirus family HERV-W.. Mol Biol Evol.

[83] Cullen BR, Lomedico PT, Ju G (1984). Transcriptional interference in avian retroviruses–implications for the promoter insertion model of leukaemogenesis.. Nature.

[84] Rabson AB, Graves BJ, Coffin JM, Hughes SH, Varmus HE (1997). Synthesis and processing of viral RNA..

[85] Hollister JD, Gaut BS (2009). Epigenetic silencing of transposable elements: a trade-off between reduced transposition and deleterious effects on neighboring gene expression.. Genome research.

[86] Martin U, Steinhoff G, Kiessig V, Chikobava M, Anssar M (1999). Porcine endogenous retrovirus is transmitted neither in vivo nor in vitro from porcine endothelial cells to baboons.. Transplant Proc.

[87] van de Lagemaat LN, Medstrand P, Mager DL (2006). Multiple effects govern endogenous retrovirus survival patterns in human gene introns.. Genome Biology.

[88] Li F, Nellaker C, Yolken RH, Karlsson H (2011). A systematic evaluation of expression of HERV-W elements; influence of genomic context, viral structure and orientation.. BMC Genomics.

[89] Jjingo D, Huda A, Gundapuneni M, Marino-Ramirez L, Jordan IK (2011). Effect of the transposable element environment of human genes on gene length and expression.. Genome biology and evolution.

[90] Peaston AE, Evsikov AV, Graber JH, de Vries WN, Holbrook AE (2004). Retrotransposons regulate host genes in mouse oocytes and preimplantation embryos.. Dev Cell.

[91] Matouskova M, Blazkova J, Pajer P, Pavlicek A, Hejnar J (2006). CpG methylation suppresses transcriptional activity of human syncytin-1 in non-placental tissues.. Exp Cell Res.

[92] Reiss D, Zhang Y, Mager DL (2007). Widely variable endogenous retroviral methylation levels in human placenta.. Nucleic Acids Res.

[93] Gimenez J, Montgiraud C, Oriol G, Pichon JP, Ruel K (2009). Comparative Methylation of ERVWE1/Syncytin-1 and Other Human Endogenous Retrovirus LTRs in Placenta Tissues.. DNA Res.

[94] Wentzensen N, Coy JF, Knaebel HP, Linnebacher M, Wilz B (2007). Expression of an endogenous retroviral sequence from the HERV-H group in gastrointestinal cancers.. Int J Cancer.

[95] Trejbalova K, Blazkova J, Matouskova M, Kucerova D, Pecnova L (2011). Epigenetic regulation of transcription and splicing of syncytins, fusogenic glycoproteins of retroviral origin.. Nucl Acids Res.

[96] Smit AFA, Hubley R, Green P (1996). RepeatMasker Open-3.0..

[97] Navarro G, Raffinot M (2002). Flexible Pattern Matching in Strings: Practical On-Line Search Algorithms for Texts and Biological Sequences.Cambridge University Press..

[98] Webber MM, Quader ST, Kleinman HK, Bello-DeOcampo D, Storto PD (2001). Human cell lines as an in vitro/in vivo model for prostate carcinogenesis and progression.. The Prostate.

[99] Bello D, Webber MM, Kleinman HK, Wartinger DD, Rhim JS (1997). Androgen responsive adult human prostatic epithelial cell lines immortalized by human papillomavirus 18.. Carcinogenesis.

[100] Irizarry RA, Hobbs B, Collin F, Beazer-Barclay YD, Antonellis KJ (2003). Exploration, normalization, and summaries of high density oligonucleotide array probe level data.. Biostatistics (Oxford, England).

[101] Johnson WE, Li C, Rabinovic A (2007). Adjusting batch effects in microarray expression data using empirical Bayes methods.. Biostatistics (Oxford, England).

[102] Storey JD, Tibshirani R (2003). Statistical significance for genomewide studies.. Proceedings of the National Academy of Sciences of the United States of America.

[103] Team RDC (2008). R: A language and environment for statistical computing..

[104] Gentleman RC, Carey VJ, Bates DM, Bolstad B, Dettling M (2004). Bioconductor: open software development for computational biology and bioinformatics.. Genome Biol.

[105] Lee BShin G (2009). CleanEST: a database of cleansed EST libraries.. Nucl Acids Res.

[106] Nickerson DA, Taylor SL, Weiss KM, Clark AG, Hutchinson RG (1998). DNA sequence diversity in a 9.7-kb region of the human lipoprotein lipase gene.. Nat Genet.

