# Supplementary figures and images for: Microarray-Based Sketches of the HERV Transcriptome Landscape
Source: PLoS One. 2012 Jun 28;7(6):e40194. doi: 10.1371/journal.pone.0040194 (PMC3386233; doi:10.1371/journal.pone.0040194)

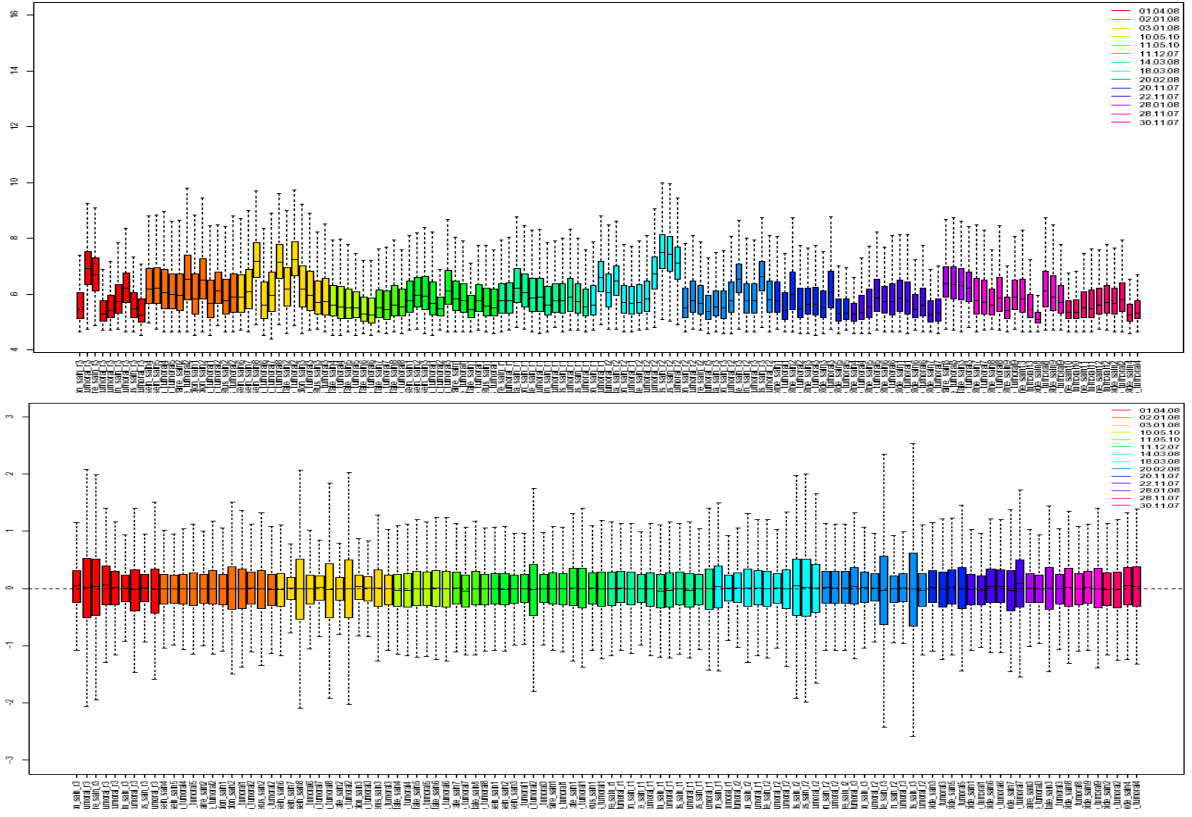

Supplement: Figure S1 — Effect of RMA-COMBAT normalization. Distribution of intensities within the dataset before (upper part) and after (lower part) RMA-COMBAT normalization. Each boxplot represents a single chip and the colors refer to experimental batches. (PDF) [file pone.0040194.s001.pdf]

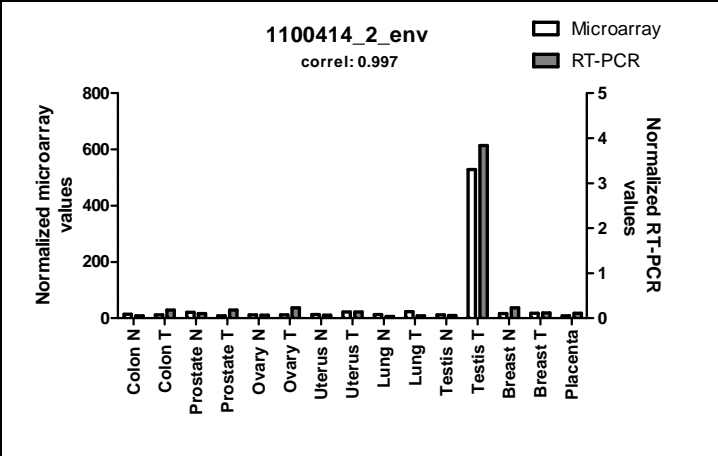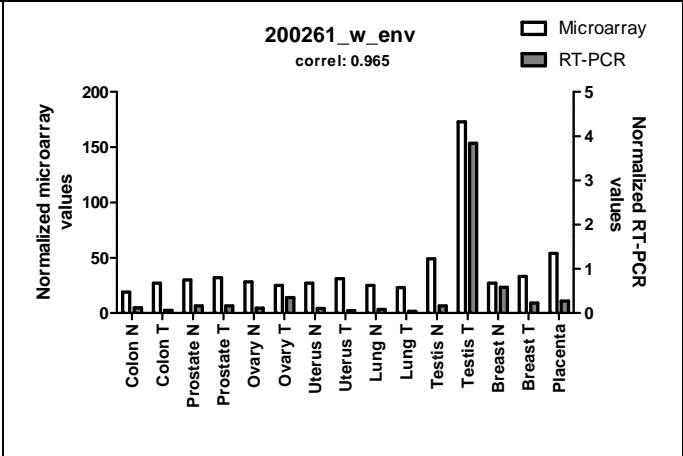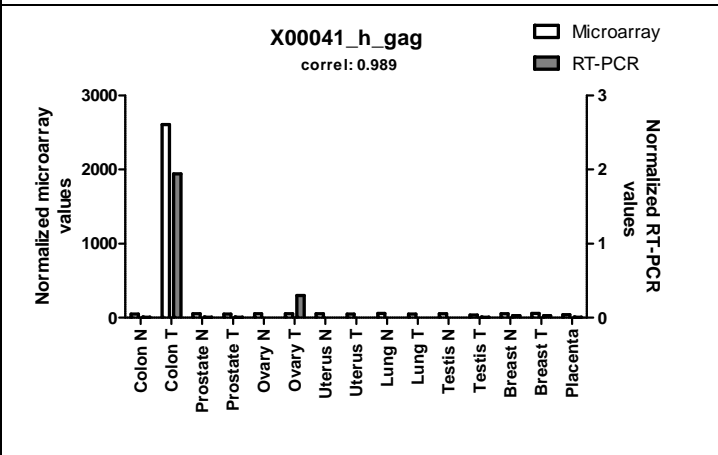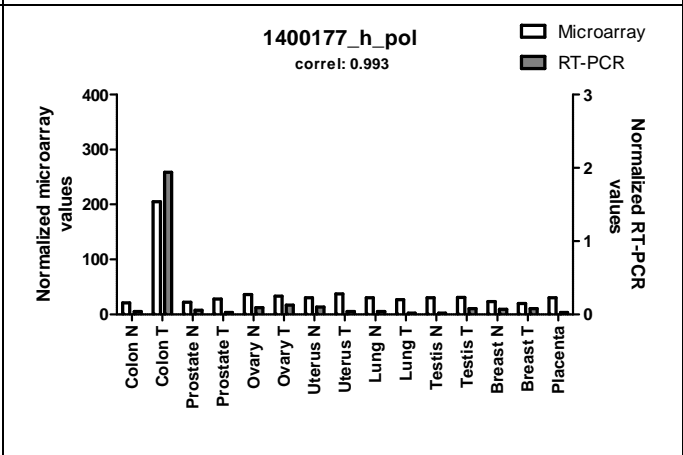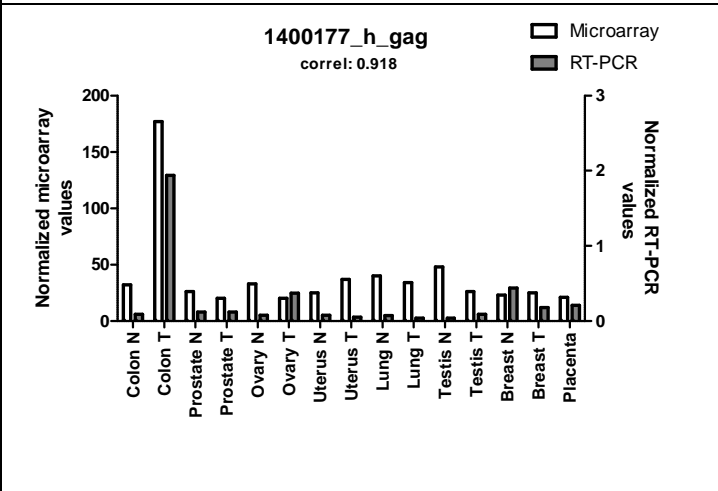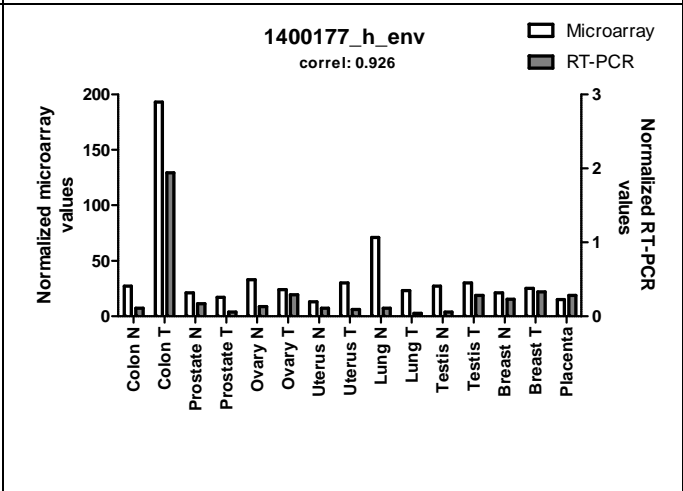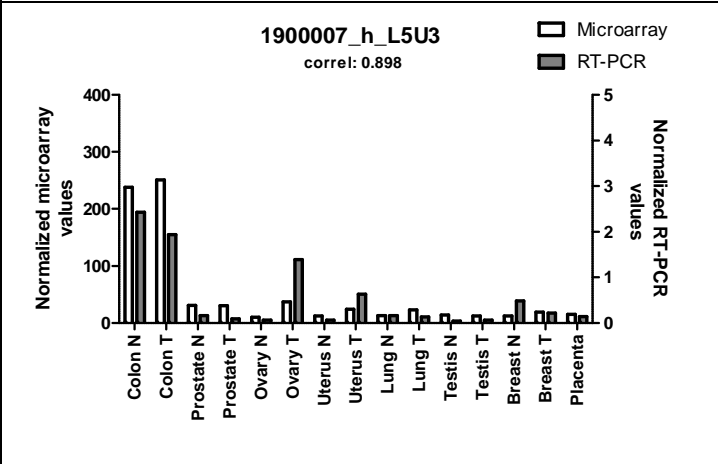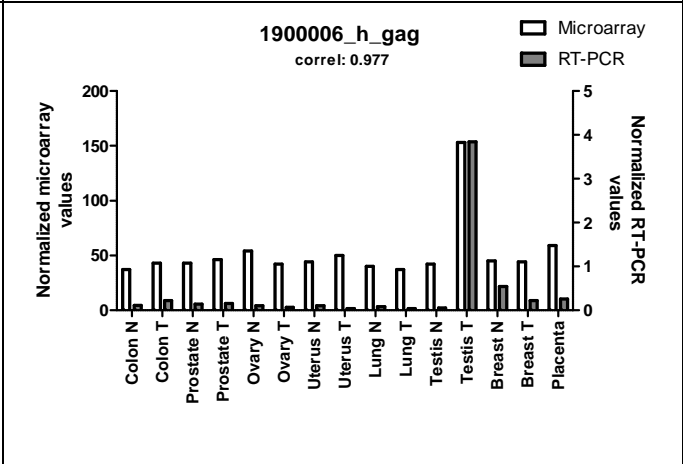

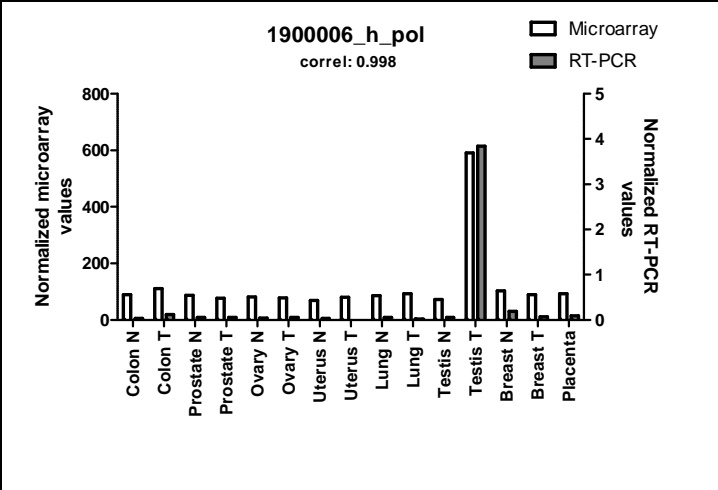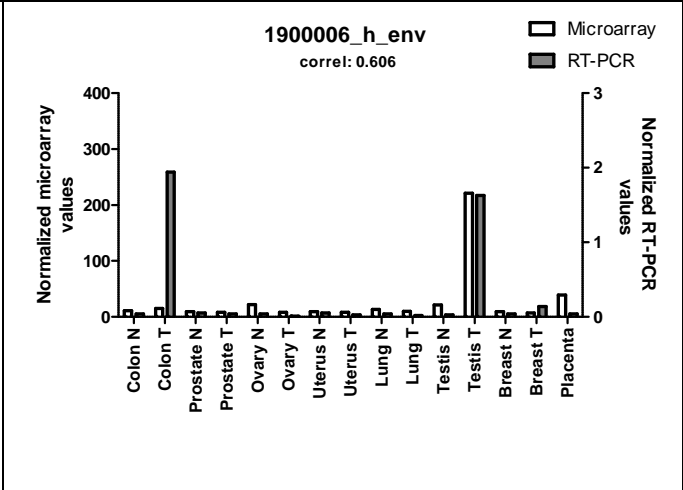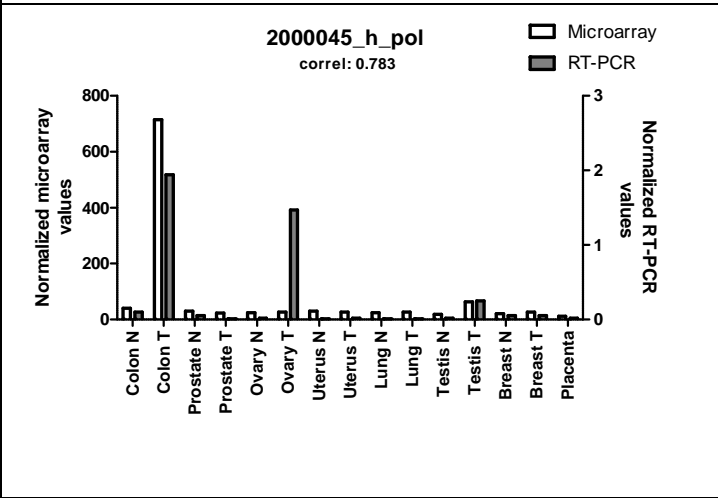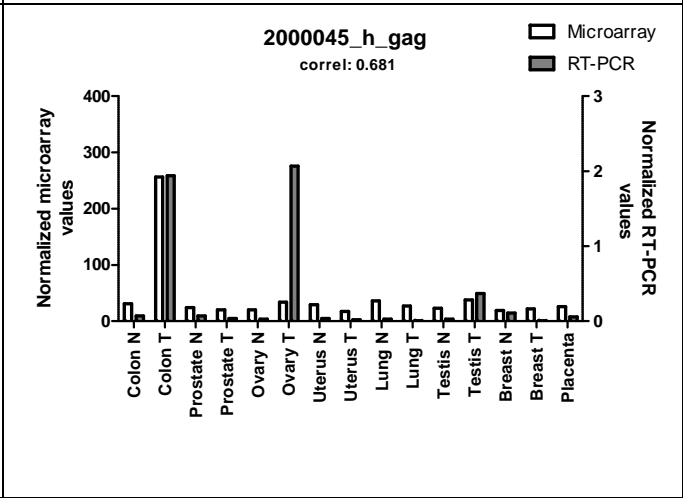

Supplement: Figure S2 — Correlations between microarray and RT-PCR results. Normalized values of microarray and RT-PCR experiments are given for 12 independent HERV sequences that belong to 8 distinct HERV loci. Correlations close to 1 indicate a strong positive linear relationship and therefore confirm the findings. Correl = Covmicroarray;RT-PCR/(sdmicroarray*sdRT-PCR). (PDF) [file pone.0040194.s002.pdf]

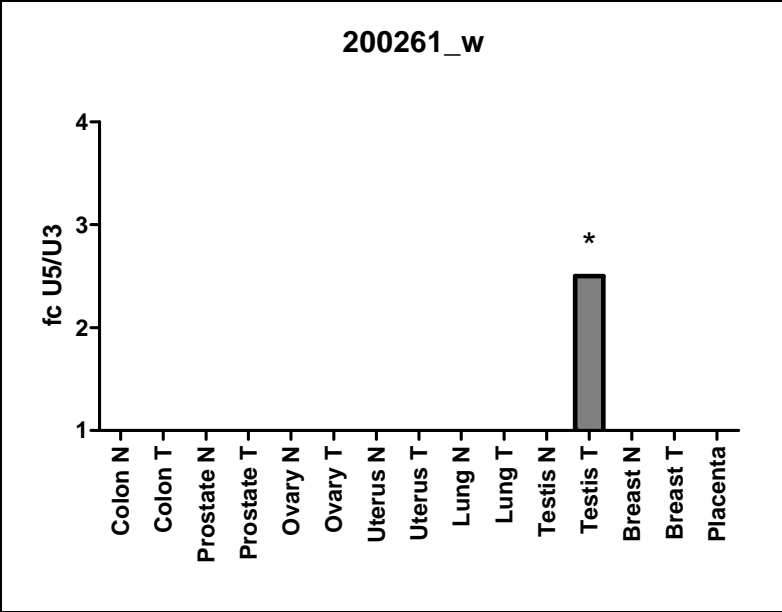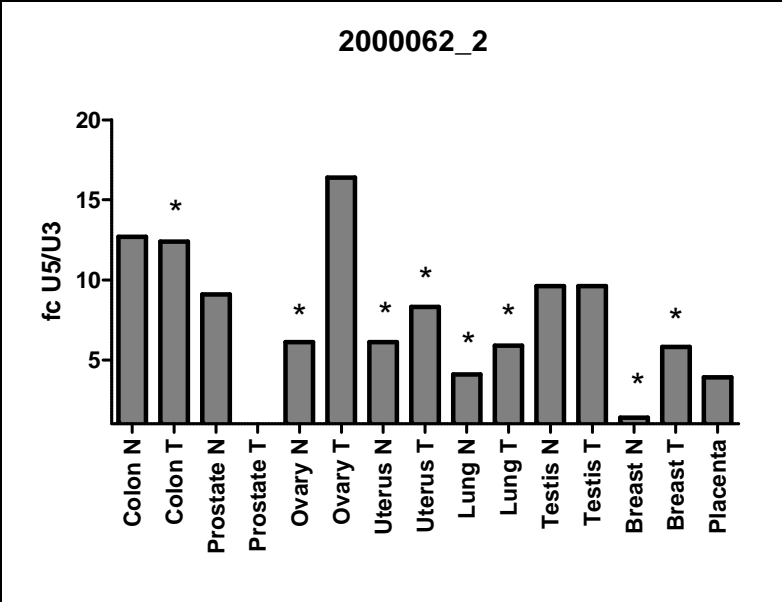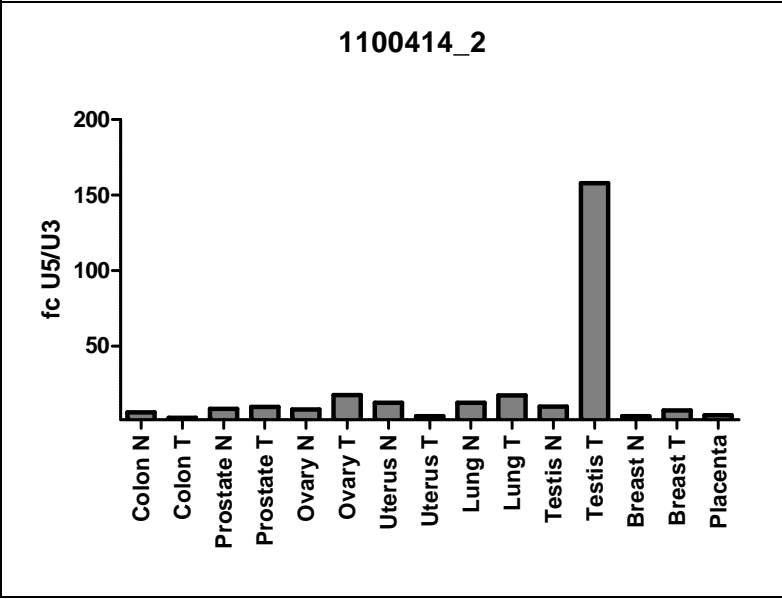

Supplement: Figure S3 — RT-PCR analyses of LTR promoter functions. The promoter activity of 3 independent LTRs was evaluated in RT-PCR. Relative expression of U5 vs U3 is given by FcU5/U3 = (EffU3 CtU3)/(EffU5 CtU5). Values greater than 1 indicate a promoter activity. An asterisk (*) highlights tissues for which the promoter activity has been unequivocally found using the microarrays. In the particular case of 1100414_2 no probeset was defined within the LTR and consequently the promoter activity in testicular tumor could not be detected using microarrays. (PDF) [file pone.0040194.s003.pdf]
