# Supplementary material for: Microarray-Based Sketches of the HERV Transcriptome Landscape
Source: PLoS One. 2012 Jun 28;7(6):e40194. doi: 10.1371/journal.pone.0040194 (PMC3386233; doi:10.1371/journal.pone.0040194)

300083_w-BG956138.jpg

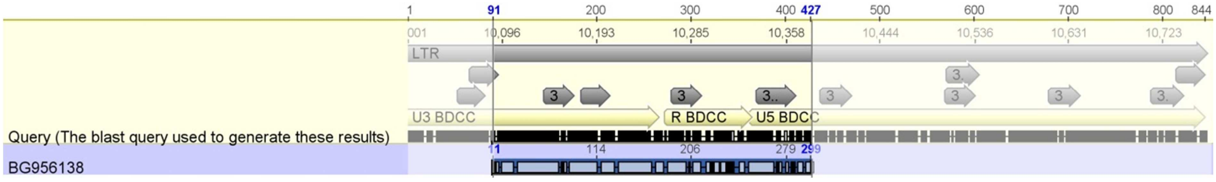

400207_w-AI738459.jpg

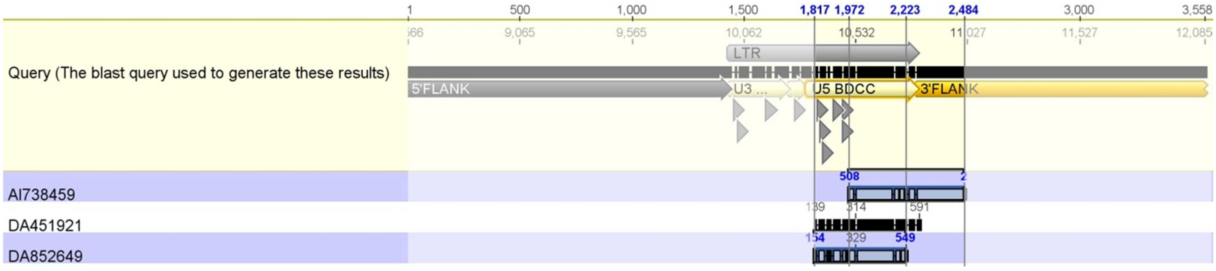

400652_w-AI915245.jpg

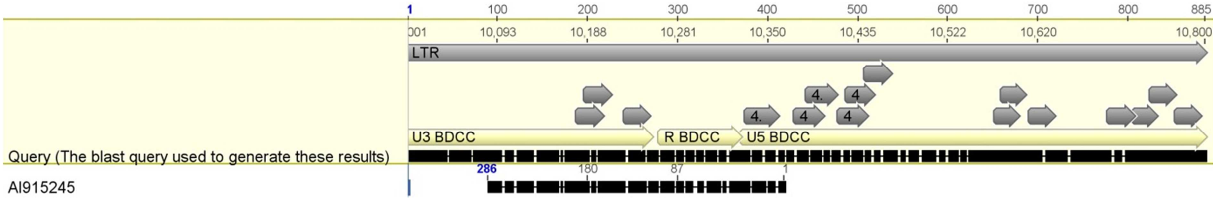

500529_w-DB092475.jpg

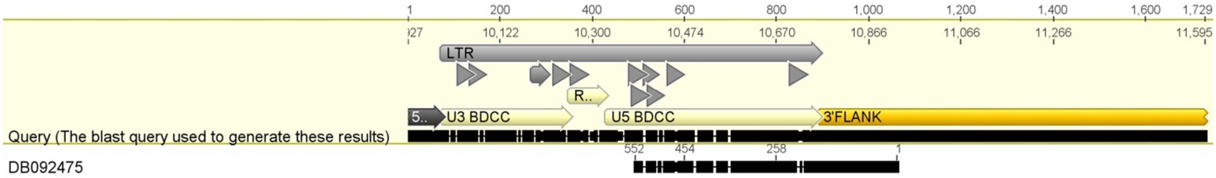

500617_w-BG014730.jpg

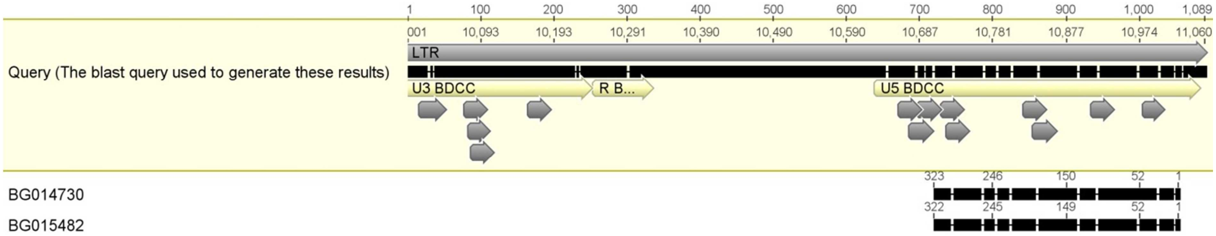

600462_w-CR745334.jpg

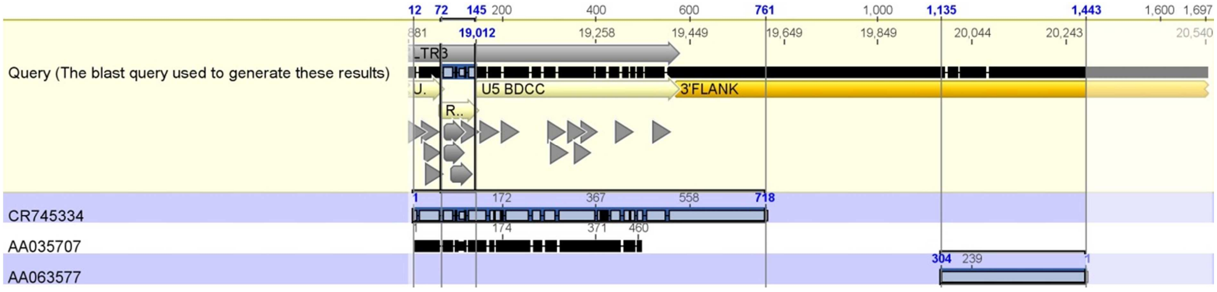

700126_w-BM723292.jpg

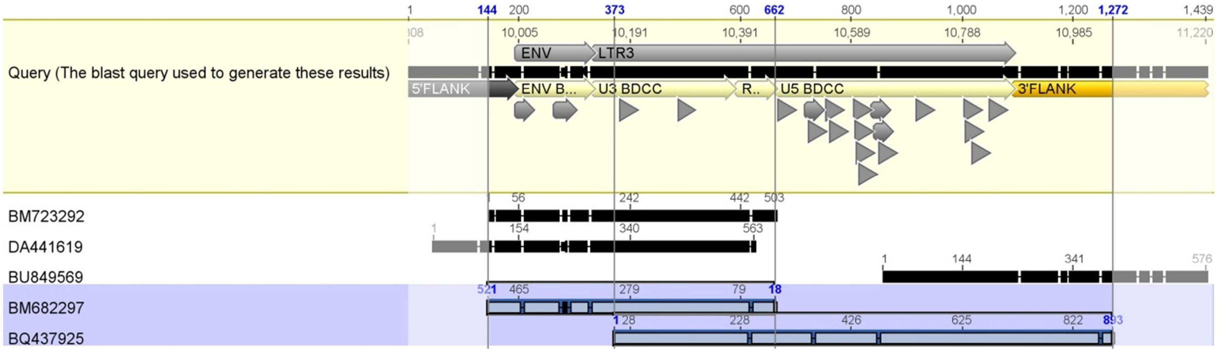

700341_w-BX357209.jpg

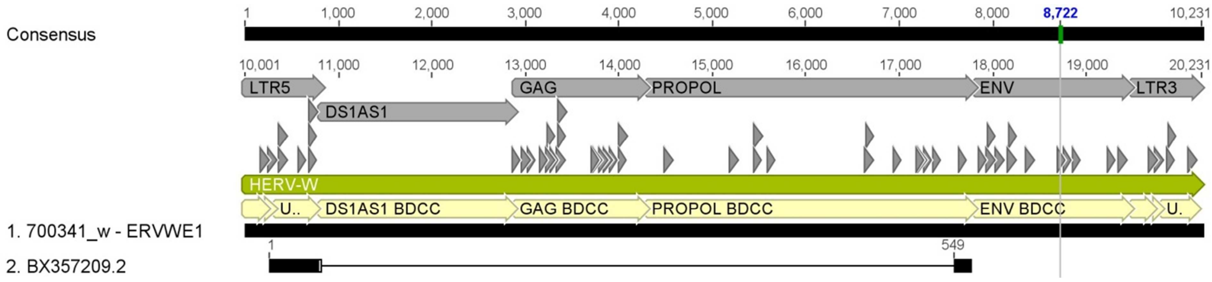

700341_w-ERVWE1_3LTR.jpg

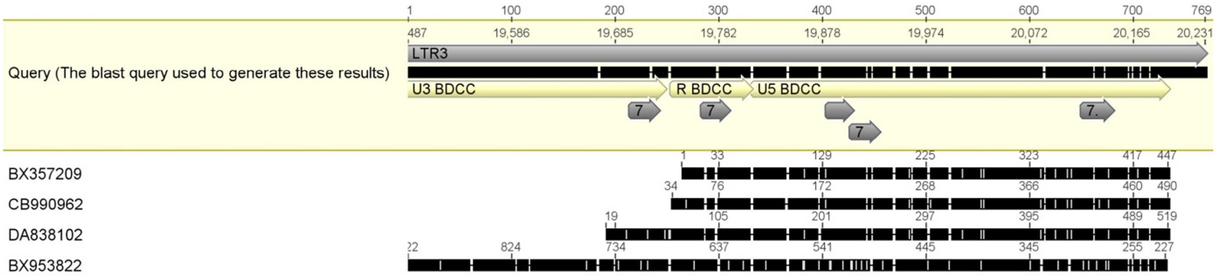

700341_w-ERVWE1_5LTR.jpg

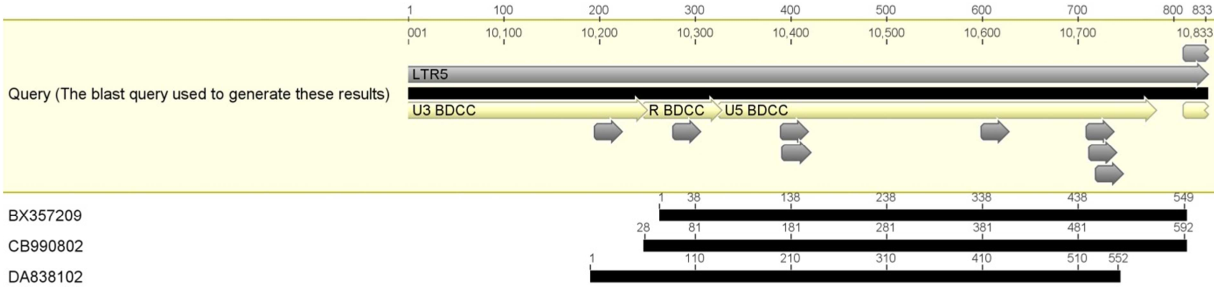

800216_w-BM510276.jpg

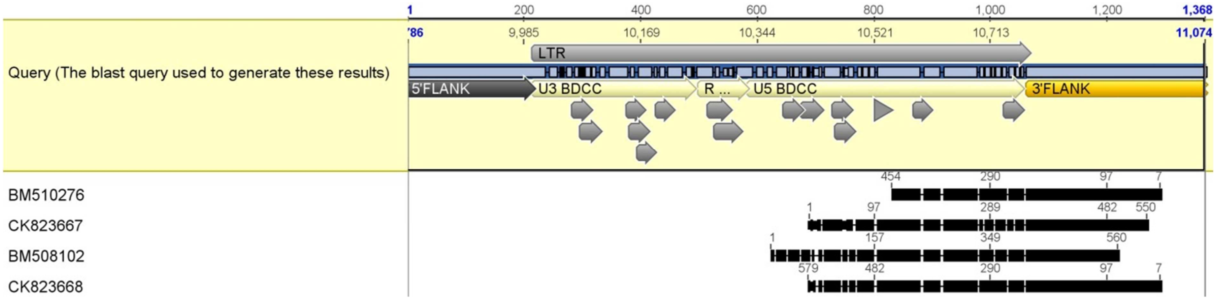

900019_w-AV721910.jpg

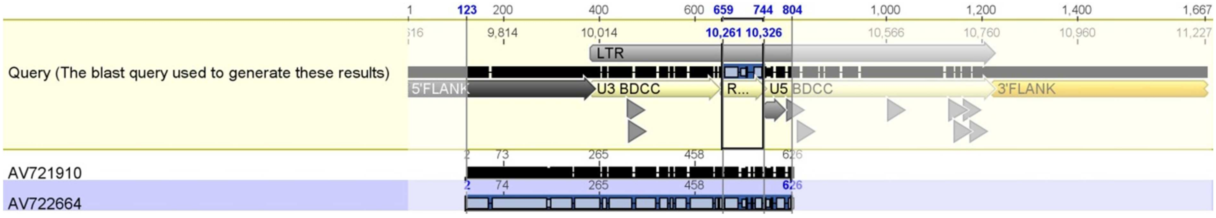

900131_w-BF591249.jpg

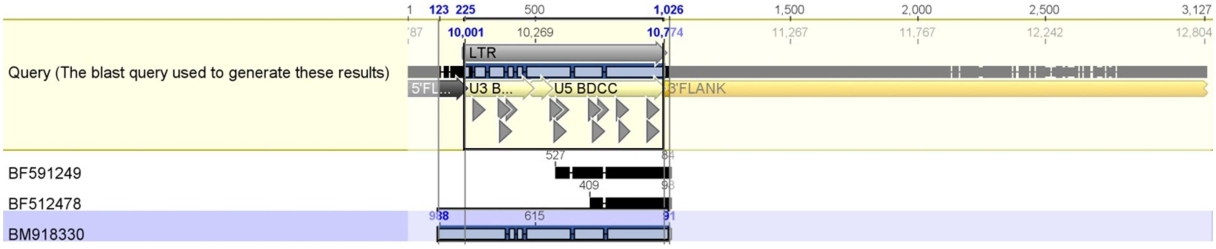

1000135_w-DA383318.jpg

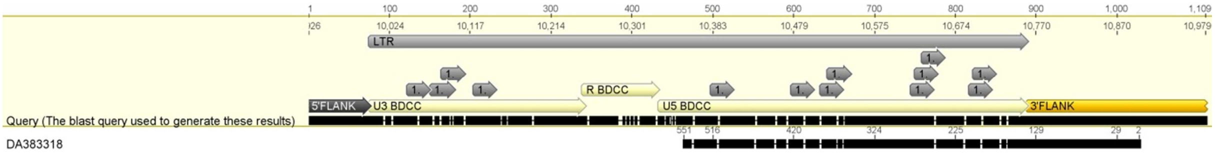

1100102_w-BU621450.jpg

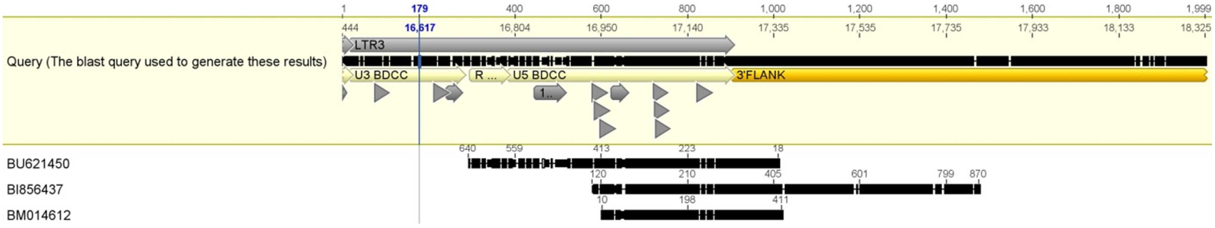

1200505_w-AA781468.jpg

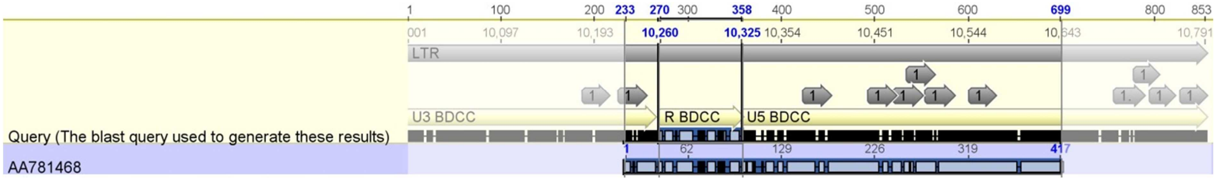

1300265_w-BQ424090.jpg

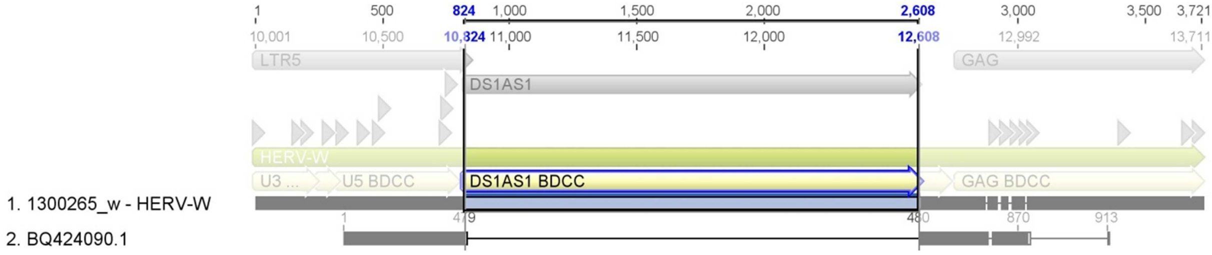

Supplement: Figure S4 — Pictures illustrating alignments of HERV-W loci with their best EST counterpart. Each alignment is designated by the name of the locus as it stands on the microarray, followed by the name of the most similar EST. The alignment explicitly states the retroviral structure including LTR U3, R and U5 subdomains, as well as flanking regions. Probes defined on the array are indicated by grey arrows. The sequence used for the query is represented as well as the EST retained for analysis, as developed in Table S7. Accession number and EST count are shown. Arbitrary blue numbering of HERV subdomain and the aligned EST together with blue vertical bars are indicated when required to facilitate the reading, e.g. clones overlapping U5 and 5′ flanking region for 400207_w-AI738459.jpg. Best score EST aligned with 5′ (700341_w-ERVWE1_5LTR.jpg) and 3′ (700341_w-ERVWE1_3LTR.jpg) LTRs of the ERVWE1 locus are included to highlight the limits of information provided by ESTs. (PDF) [file pone.0040194.s004.pdf]
