# Supplementary material for: Microarray-Based Sketches of the HERV Transcriptome Landscape
Source: PLoS One. 2012 Jun 28;7(6):e40194. doi: 10.1371/journal.pone.0040194 (PMC3386233; doi:10.1371/journal.pone.0040194)
Supplement: Table S1 — HERV prototypes used for the construction of HERV-gDB3. Accession numbers, genomic localizations and the limits of the functional region within the prototype sequences (U3, R, U5, gag, pol env) are given for the 6 HERV families studied. a for HERV identification and gene cutting out. b for LTR sub region cutting out. (PDF) [file pone.0040194.s005.pdf]

HERV prototype	# accession of reference sequence	Genomic position	U3	R	U5	gag	pol	env
HERV-W	AC007566	(-) chr 7: 91935220-91945441	1-247	248-326	327-780	2887-4311	4312-7811	7812-9486
HERV-H	AC009495 [a]	(-) chr 2: 166272306-166280954				1257-1827	2404-5957	6255-8009
	AJ289709 [b]		1-381	382-443	444-483			
HERV-E 4.1	M10976	(-) chr 19: 20721466-20730278	1-424	425-451	452-495	1043-2652	2653-6216	6239-8254
HERV-FRD	AC004022	(+) chr 7: 94819839-94819943	1-493	495-607	608-714	728-1936	4771-8279	8280-9886
HERV-K HML-2	Y17832	(-) chr 7: 4588583-4598054	1-560	561-813	814-968	1112-3112	4130-6748	6465-8549
HERV-K HML-5	AC004536	(+) chr 6: 117541379-117549142	1-280	281-323	324-482	635-1460	2792-5355	5381-7105
