# Supplementary material for: Microarray-Based Sketches of the HERV Transcriptome Landscape
Source: PLoS One. 2012 Jun 28;7(6):e40194. doi: 10.1371/journal.pone.0040194 (PMC3386233; doi:10.1371/journal.pone.0040194)
Supplement: Table S2 — Biological samples included in the study. List of biological samples included in the study (samples) and used in the composition of analysis groups (set of samples). Information on pathological status, age and sex are provided when available. Matched tumoral/normal samples are indicated (paired with). An asterisk (*) highlights samples that were not used for the microarray study. (PDF) [file pone.0040194.s006.pdf]

set of samples	samples	clinical status	age/sex	paired with	supplier
colon N	colon N1	Adjacent normal	40/M	colon T1	Life Technologies
	colon N2	Adjacent normal	62/F	colon T2	Clinisciences
	colon N3	Adjacent normal	68/F	colon T3	Clinisciences
	colon N4	Adjacent normal	62/F	colon T4	Clinisciences
colon T	colon T1	Adenocarcinoma	40/M	colon N1	Life Technologies
	colon T2	Adenocarcinoma, mucinous moderately differentiated	62/F	colon N2	Clinisciences
	colon T3	Adenocarcinoma, mucinous poorly differentiated	68/F	colon N3	Clinisciences
	colon T4	Adenocarcinoma, moderately differentiated	62/F	colon N4	Clinisciences
lung N	lung N1	Adjacent normal	72/M	lung T1	Life Technologies
	lung N2	Adjacent normal	58/M	lung T2	CRB Nancy
	lung N3	Adjacent normal	67/M	lung T3	CRB Nancy
	lung N4	Adjacent normal	53/M	lung T4	CRB Nancy
	lung N5	Adjacent normal	56/M	lung T5	CRB Nancy
	lung N6	Adjacent normal	58/M	lung T6	CRB Nancy
	lung N7	Adjacent normal	52/M	lung T7	CRB Nancy
	lung N8	Adjacent normal	47/M	lung T8	CRB Nancy
	lung N9	Adjacent normal	78/M	lung T9	CRB Nancy
	lung N10	Adjacent normal	63/M	lung T10	CRB Nancy
lung T	lung T1	Squamous cell carcinoma	72/M	lung N1	Life Technologies
	lung T2	Epidermoid, T1N0	58/M	lung N2	CRB Nancy
	lung T3	Epidermoid, T1N0	67/M	lung N3	CRB Nancy
	lung T4	Epidermoid, T2N0	53/M	lung N4	CRB Nancy
	lung T5	Epidermoid, T1N0	56/M	lung N5	CRB Nancy
	lung T6	Epidermoid, T2N1	58/M	lung N6	CRB Nancy
	lung T7	Epidermoid, T2N1	52/M	lung N7	CRB Nancy
	lung T8	Epidermoid, T2N0	47/M	lung N8	CRB Nancy
	lung T9	Epidermoid, T1N0	78/M	lung N9	CRB Nancy
	lung T10	Epidermoid, T2N1	63/M	lung N10	CRB Nancy
breast N	breast N1	Adjacent normal	56/F	breast T1	Clinisciences
	breast N2	Adjacent normal	41/F	breast T2	Clinisciences
	breast N3	Adjacent normal	63/F	breast T3	Clinisciences
	breast N4	Adjacent normal	50/F	breast T4	Clinisciences
	breast N5	Adjacent normal	50/F	breast T5	Clinisciences
	breast N6	Adjacent normal	47/F	breast T6	Clinisciences
	breast N7	Adjacent normal	53/F	breast T7	Clinisciences
	breast N8	Adjacent normal	58/F	breast T8	Clinisciences
breast T	breast T1	Invasive ductal carcinoma	56/F	breast N1	Clinisciences
	breast T2	Invasive ductal carcinoma	41/F	breast N2	Clinisciences
	breast T3	Invasive ductal carcinoma	63/F	breast N3	Clinisciences
	breast T4	Invasive ductal carcinoma	50/F	breast N4	Clinisciences
	breast T5	Invasive ductal carcinoma	50/F	breast N5	Clinisciences
	breast T6	Invasive ductal carcinoma	47/F	breast N6	Clinisciences
	breast T7	Invasive lobular carcinoma, moderately differentiated	53/F	breast N7	Clinisciences
	breast T8	Invasive ductal carcinoma, poorly differentiated	58/F	breast N8	Clinisciences
ovary N	ovary N1	Adjacent normal	32/F	ovary T1	Life Technologies
	ovary N2	Adjacent normal	71/F	ovary T2	Clinisciences
	ovary N3	Adjacent normal	20/F	ovary T3	Clinisciences
	ovary N4	Adjacent normal	40/F	ovary T4	Clinisciences
ovary T	ovary T1	Papillary cystadeno carcinoma	32/F	ovary N1	Life Technologies
	ovary T2	Right ovary endometrioid carcinoma, moderately differentiated	71/F	ovary N2	Clinisciences
	ovary T3	Yolk sac cystadenoma	20/F	ovary N3	Clinisciences
	ovary T4	Mucinous cystodeno carcinoma	40/F	ovary N4	Clinisciences
prostate N	prostate N1	Adjacent normal	82/M	prostate T1	Clinisciences
	prostate N2	Adjacent normal	53/M	prostate T2	Life Technologies
	prostate N3	Adjacent normal	73/M	prostate T3	Life Technologies
	prostate N4	Adjacent normal	56/M	prostate T4	CH Lyon Sud
	prostate N5	Adjacent normal	63/M	prostate T5	CH Lyon Sud
	prostate N6	Adjacent normal	73/M	prostate T6	CH Lyon Sud
	prostate N7	Adjacent normal	66/M	prostate T7	CH Lyon Sud
	prostate N8	Adjacent normal	68/M	prostate T8	CH Lyon Sud
prostate T	prostate T1	Prostate cancer	82/M	prostate N1	Clinisciences
	prostate T2	Adenocarcinoma, well differentiated	53/M	prostate N2	Life Technologies
	prostate T3	Adenocarcinoma, well to poorly differentiated Gleason 7	73/M	prostate N3	Life Technologies
	prostate T4	Prostate adenocarcinoma, pT3bN1 Gleason 7	56/M	prostate N4	CH Lyon Sud
	prostate T5	Prostate adenocarcinoma, pT2cN0 Gleason 7	63/M	prostate N5	CH Lyon Sud
	prostate T6	Prostate adenocarcinoma, pT2cN0 Gleason 6	73/M	prostate N6	CH Lyon Sud
	prostate T7	Prostate adenocarcinoma, pT2cN0 Gleason 7	66/M	prostate N7	CH Lyon Sud
	prostate T8	Prostate adenocarcinoma, pT2cN0 Gleason 7	68/M	prostate N8	CH Lyon Sud
testis N	testis N1	Adjacent normal	33/M	testis T1	Life Technologies
	testis N2 *	Adjacent normal	26/M	testis T2	Life Technologies
	testis N3 *	Adjacent normal	30/M	testis T3	Clinisciences
testis T	testis T1	Seminoma	33/M	testis N1	Life Technologies
	testis T2 *	Seminoma, T3N1	26/M	testis N2	Life Technologies
	testis T3 *	Seminoma	30/M	testis N3	Clinisciences
uterus N	uterus N1	Adjacent normal	47/F	uterus T1	Life Technologies
	uterus N2	Adjacent normal	49/F	uterus T2	Clinisciences
	uterus N3	Adjacent normal	55/F	uterus T3	Clinisciences
	uterus N4	Adjacent normal	41/F	uterus T4	Clinisciences
uterus T	uterus T1	Choriocarcinoma	47/F	uterus N1	Life Technologies
	uterus T2	Adenocarcinoma, poorly differentiated	49/F	uterus N2	Clinisciences
	uterus T3	Adenocarcinoma, poorly differentiated	55/F	uterus N3	Clinisciences
	uterus T4	Endometrioid adenocarcinoma	41/F	uterus N4	Clinisciences
placenta	placenta N1	Normal term	30/F	no	Life Technologies
