# Supplementary material for: Microarray-Based Sketches of the HERV Transcriptome Landscape
Source: PLoS One. 2012 Jun 28;7(6):e40194. doi: 10.1371/journal.pone.0040194 (PMC3386233; doi:10.1371/journal.pone.0040194)
Supplement: Table S5 — Matching of tissue-specific HERV sequences with Expressed Sequenced Tag (EST) databases. The CleanEST database [105] was used to retrieve ESTs associated with tissues of interest in order to construct 6 reference EST groups: colon (311122 ESTs), lung (441913 ESTs), ovary (123944 ESTs), placenta (321881 ESTs), prostate (69860 ESTs) and testis (264243 ESTs). Each EST group was blasted against the HERV sequences composing the expression profiles shown in Figure 1, following the procedure detailed in the EST analysis part of the materials and methods section. Hits were normalized by the total number of HERV loci of the expression profile and by the total number of ESTs forming the reference group. The ranking of the value is associated with a color code highlighting the enrichment of tissue-associated ESTs: green (1/6), yellow (2/6) and red (>2/6). (PDF) [file pone.0040194.s009.pdf]

colon T

EST	*Normalized hits*
colon	65,7
lung	39,3
ovary	35,6
placenta	21,0
prostate	11,7
testis	28,2

ovary T

EST	*Normalized hits*
colon	48,0
lung	55,0
ovary	58,8
placenta	28,4
prostate	35,8
testis	43,0

placenta N

EST	*Normalized hits*
colon	12,0
lung	14,8
ovary	16,9
placenta	20,6
prostate	7,8
testis	17,9

planceta N + testis T

EST	*Normalized hits*
colon	17,7
lung	16,1
ovary	32,3
prostate	16,1
placenta + testis	48,0

testis N

EST	*Normalized hits*
colon	44,1
lung	39,2
ovary	31,4
placenta	27,5
prostate	34,6
testis	40,5

testis T

EST	*Normalized hits*
colon	17,6
lung	13,3
ovary	10,0
placenta	10,6
prostate	7,3
testis	14,6

lung N

EST	*Normalized hits*
colon	53,8
lung	46,7
ovary	51,4
placenta	33,8
prostate	57,3
testis	62,0

prostate T

EST	*Normalized hits*
colon	17,1
lung	13,6
ovary	22,9
placenta	15,8
prostate	20,3
testis	22,4
