# Supplementary material for: Microarray-Based Sketches of the HERV Transcriptome Landscape
Source: PLoS One. 2012 Jun 28;7(6):e40194. doi: 10.1371/journal.pone.0040194 (PMC3386233; doi:10.1371/journal.pone.0040194)
Supplement: Table S6 — Primers used for RT-PCR experiments. Forward and reverse primer sequences used for RT-PCR analyses. The Tm of each primer pair was determined as described in the related materials and methods section. The domain of application is indicated (normalization, tropism, promoter function). (PDF) [file pone.0040194.s010.pdf]

Target	Tm (°C)	Forward primer (5' to 3')	Reverse primer (5' to 3')	Application
G6PD	55	TGCAGATGCTGTGTCTGG	CGTACTGGCCCAGGACC	normalization
GAPDH	55	GAAGGTGAAGGTCGGAGTC	GAAGATGGTGATGGGATTTC	normalization
HPRT	55	GTGATGATGAACCAGGTTATGACCTTG	CTACAGTCATAGGAATGGATCTATCAC	normalization
1100414_2_env	57	GGCCTATGTGCCTTTCCCGCC	TCCTGGTGCTGTCCCTAAGCAA	tropism
1100414_2_L5U3	59	AGCTCAACAAACACTTCCATGCTCA	TCCTGCACCGCCCTTAATCCATTCA	promoter function
1100414_2_L5U5	57	TGGTTCCCCGGTTCCCCTTATTTCT	CCTACACACCTGTGGGTGTTTCTCA	promoter function
200261_w_env	57	TTGTCTCTTCTGGAATCAAAGCTGC	TGCAATTGAGATTTCCTCGGGAGGG	tropism
200261_w_L5U3	61	GCTCACACCCAACCAATCAGAGAGC	CTGGAAGGCCTGGGTTTATATCGCA	promoter function
200261_w_L5U5	55	ATAACACTCACCTGGCATGGCCCAG	ACTTCCAAGATGGTGGCTAGCAGCT	promoter function
X00041_h_gag	61	CAGGCGTTGCTGAGTGTGTCTAATC	TGGAGCCTGAGGAAGAATTGGGACC	tropism
1400177_h_pol	55	CACGGTAGAAGGTAGTAAGCGCGTC	GCGGCAATGAGATGTGGCTGTAGTC	tropism
1400177_h_gag	61	TTCCTAGTCTTTGTCCCCAATGCAA	AGGTGTGAGGAGGCGAGGTGATAAA	tropism
1400177_h_env	61	TCCAAAACCATATGCAGTCCATCAC	AGCTGAAGGGAGGTCTTGTGGTAAG	tropism
1900007_h_L5U3	57	ATATCCCCTACGACCGGCTCATATA	TGGGGCAGAAACAGATCACAATGGT	tropism
1900006_h_gag	59	GGTGCCTGAAGTCCATCAGTCCCTT	GCAGGAGCAGAGGCTGAGGAAGAAT	tropism
1900006_h_pol	65	TCCCCAGCCCAAAGCTTCCTTCACG	GCGTTGAGCGGGGTAAGGGTGATTA	tropism
1900006_h_env	61	CCACCGAGGCCTTGACTGACT	GGGAGGGCCCAGGACATCCAA	tropism
2000045_h_pol	59	TACAGCATGGGCACCTATAAACTCT	TAAGTGAAGGCAAAGAGAGGCTGGG	tropism
2000045_h_gag	59	CCCAAGCGGCGCTGAGTCTT	TGGGATGAAGGGAGGGGAGGC	tropism
2000062_2_L5U3	55	TGCTGGCAGAAACAAGTGCTGTGTT	GGTTCCTGTGGCCTCCTGCAGTGAT	promoter function
2000062_2_L5U5	63	GCGTGGGTCCTCCCTATGCTGAGC	AAGGGTGGCCAGCTGCTCCACACCT	promoter function
